# A Comparison of a Pulse-Based Diet and the Therapeutic Lifestyle Changes Diet in Combination with Exercise and Health Counselling on the Cardio-Metabolic Risk Profile in Women with Polycystic Ovary Syndrome: A Randomized Controlled Trial

**DOI:** 10.3390/nu10101387

**Published:** 2018-09-30

**Authors:** Maryam Kazemi, Laura E. McBreairty, Donna R. Chizen, Roger A. Pierson, Philip D. Chilibeck, Gordon A. Zello

**Affiliations:** 1 Division of Nutrition and Dietetics, College of Pharmacy and Nutrition, 104 Clinic Place, University of Saskatchewan, Saskatoon, SK S7N 2Z4, Canada; maryam.kazemi@usask.ca (M.K.); l.e.mcbreairty@gmail.com (L.E.M.); 2 Obstetrics and Gynecology, College of Medicine, University of Saskatchewan, 103 Hospital Drive, Saskatoon, SK S7N 0W8, Canada; donna.chizen@usask.ca (D.R.C.); roger.pierson@usask.ca (R.A.P.); 3 College of Kinesiology, Physical Activity Complex, University of Saskatchewan, 87 Campus Drive, Saskatoon, SK S7N 5B2, Canada; phil.chilibeck@usask.ca

**Keywords:** insulin, glucose, lifestyle, lipid, metabolic syndrome, physical activity, pulse foods, dietary intake

## Abstract

We compared the effects of a low-glycemic index pulse-based diet, containing lentils, beans, split peas, and chickpeas, to the Therapeutic Lifestyle Changes (TLC) diet on cardio-metabolic measures in women with polycystic ovary syndrome (PCOS). Ninety-five women (18–35 years) enrolled in a 16-week intervention; 30 women in the pulse-based and 31 in the TLC groups completed the study. Women participated in aerobic exercise training (minimum 5 days/week for 45 min/day) and were counselled (monthly) about PCOS and lifestyle modification. Women underwent longitudinal follow-up post-intervention. The pulse-based group had a greater reduction in total area under the curve for insulin response to a 75-g oral glucose tolerance test (mean change ± SD: −121.0 ± 229.9 vs. −27.4 ± 110.2 µIU/mL × min; *p* = 0.05); diastolic blood pressure (−3.6 ± 6.7 vs. −0.2 ± 6.7 mmHg, *p* = 0.05); triglyceride (−0.2 ± 0.6 vs. 0.0 ± 0.5 mmol/L, *p* = 0.04); low-density lipoprotein cholesterol (−0.2 ± 0.4 vs. −0.1 ± 0.4 mmol/L, *p* = 0.05); total cholesterol/high-density lipoprotein cholesterol (TC/HDL-C; −0.4 ± 0.4 vs. 0.1 ± 0.4, *p* < 0.001); and a greater increase in HDL-C (0.1 ± 0.2 vs. −0.1 ± 0.2 mmol/L, *p* < 0.01) than the TLC group. Decreased TC/HDL-C (*p* = 0.02) at six-month and increased HDL-C and decreased TC/HDL-C (*p* ≤ 0.02) at 12-month post-intervention were maintained in the pulse-based group. A pulse-based diet may be more effective than the TLC diet at improving cardio-metabolic disease risk factors in women with PCOS. Trial registration: CinicalTrials.gov identifier, NCT01288638.

## 1. Introduction

Polycystic ovary syndrome (PCOS) is a prevalent endocrinopathy and the leading cause of anovulatory infertility among reproductive-age women worldwide, with a prevalence of up to 18% [1,2]. Women with PCOS exhibit profound metabolic abnormalities including peripheral insulin resistance (IR) and compensatory hyperinsulinemia, impaired glucose metabolism, dyslipidemia, hypertension, and abdominal adiposity [3,4]. These features are components of metabolic syndrome (MetS), which is defined as a complex of five interrelated risk factors for cardiovascular disease (CVD) and type 2 diabetes (DM2) [5]. Compared to other clinical presentations associated with PCOS, metabolic aberrations represent critical contributors to increased rates of long-term morbidity and mortality in women with PCOS [2,4].

Lifestyle modifications comprised of dietary, exercise and behavioral therapies are recommended as first-line approaches in the management of PCOS [6,7,8,9]. However, according to the recent international guidelines for the assessment and management of PCOS currently there is “no or limited evidence” about a specific dietary composition which is better than the other to improve PCOS health outcomes [10]. The most favorable dietary composition to facilitate metabolic changes in women with PCOS remains controversial [10,11]. Previous randomized controlled trials comparing a Dietary Approaches to Stop Hypertension eating plan with a control diet [12,13], a high protein with a normal protein diet [14], and a high protein to a high carbohydrate diet [15] have shown minimal differences in the majority of PCOS health outcomes. Rather, the overall finding was that weight loss had a positive effect on PCOS outcomes regardless of dietary composition. Rationalized by the interplay between IR and obesity, work to date has provided insights into the short-term benefits of energy restriction coupled with low-glycemic index (GI) diets on increasing insulin sensitivity, improving glucose regulation, and inducing weight loss in overweight and obese women with PCOS [16,17,18]. However, energy-restricted dietary interventions are limited in terms of sustainability in the long-term and applicability to women with PCOS across body mass index (BMI) and PCOS phenotypes. A paucity of evidence exists on an optimal dietary composition without energy restriction on cardio-metabolic aberrations associated with PCOS.

A pulse-based diet has the potential to modulate metabolic aberrations associated with PCOS. Pulses, that is, dried edible seeds of the legume family including split-peas, dry beans, lentils, and chickpeas, are high in fiber, contain complex carbohydrates with a low GI, are low in fat, contain high-quality protein, have low sodium content, and are a significant source of vitamins and minerals, such as iron, zinc, folate, calcium, magnesium, and potassium [19]. Long-term consumption of pulses in other populations, who share metabolic abnormalities with women with PCOS, has been associated with positive metabolic effects such as lowering postprandial blood glucose and insulin concentrations, and decreasing hypercholesterolemia, blood pressure, and obesity [20,21,22,23]. The TLC diet, endorsed by the National Cholesterol Education Program (NCEP) Adult Treatment Panel III, is an integral component of a non-pharmacologic healthful lifestyle habits program designed to decrease low-density lipoprotein cholesterol (LDL-C) concentrations in individuals with hypercholesterolemia. The TLC diet is a nutritionally balanced diet, resulting in increasing fiber consumption, decreasing saturated fat and dietary cholesterol intakes, as well as adding LDL-C lowering dietary options such as viscous fiber and plant stanol/sterol esters [24]. The TLC diet was considered, for our study, as a control healthy diet with the potential to improve PCOS cardio-metabolic disruptions.

Physical activity and behavioral modification strategies, including structured educational programs, problem-solving, and self-management have been recommended as integral components of comprehensive, successful, and sustainable lifestyle change programs for women with PCOS [10]. Aerobic exercise training, education, and health counselling about PCOS and lifestyle management were included as standard of care for women with PCOS. Face-to-face health counselling was provided by an interdisciplinary team of health professionals to establish educated behavioral change techniques, engagement, self-monitoring, motivation, and social support of our participants. 

We compared the effect of a pulse-based diet to the NCEP TLC diet on cardio-metabolic disease risk measures in reproductive-age women with PCOS, for 16 weeks without energy restriction, when the diets were combined with aerobic exercise and health counselling. Further, our study determined the long-term effects of the intervention on the cardio-metabolic profile of women with PCOS by follow-up of the participants 12 months after the intervention. We hypothesized that, without an energy-restricted protocol, a nutritionally balanced pulse-based diet would be more effective than the TLC diet in decreasing IR and hyperinsulinemia; thereby improving cardio-metabolic sequelae associated with PCOS where both intervention groups were encouraged to exercise regularly and received health counselling.

## 2. Materials and Methods

The protocol of the study has been published [25]. The methodology is briefly summarized below.

### 2.1. Study Design

A multi-disciplinary, single-blind, metformin-stratified, parallel group randomized controlled trial design was conducted between April 2011 and June 2016 with women diagnosed with PCOS, in Saskatoon, SK, Canada. The trial protocol was approved by the Biomedical Research Ethics Board at the University of Saskatchewan. Before participants were enrolled in the study, consent was obtained in writing. All procedures were conducted in compliance with the World Medical Association Declaration of Helsinki, the Guidelines of the International Conference on Harmonization on Good Clinical Practice, and the Canadian Tri-Council Policy Statement on the Ethical Conduct for Research Involving Humans [26]. We adhered to the Consolidated Standards of Reporting Trials (CONSORT) guidelines for reporting on randomized clinical trials (See Supplementary Materials) [19]. The trial was registered at ClinicalTrials.gov (https://ClinicalTrials.gov/, NCT01288638. Lifestyle Intervention for Polycystic Ovary Syndrome: Pulse-Based Diet and Exercise).

After informed consents were obtained, women were included for the screening of PCOS and assessed for the likelihood of having of PCOS if they met the following inclusion criteria: Between 18 to 35 years of age, had irregular periods, unwanted male-pattern facial and/or body hair growth, and infertility. Women who took medications that are known or suspected to interfere with cardio-metabolic and reproductive function, weight, and/or appetite were excluded including hormonal and/or fertility medications within the past three months before recruitment; weight and appetite-affecting medications; cardiovascular disease medications, and anti-seizure or anti-psychotic medications use because of the potential to induce IR and polycystic appearing ovaries. Women who used metformin were included and were stratified to be randomized separately than women who were not using metformin. Women with untreated hyperprolactinemia or thyroid disease; or, excessive adrenal androgen production, due to congenital adrenal hyperplasia, Cushing’s syndrome, or an adrenal tumor were excluded. Further, women with medical (e.g., cardio-pulmonary) or dietary conditions that limited physical activity or consumption of a pulse-based diet (allergies or intolerances) were excluded.

Women were randomly allocated to receive either a pulse-based diet or the TLC diet after exclusion criteria were applied. Randomization was carried out using a computer-generated allocation schedule performed by an investigator who was not involved in obtaining, entering, or analyzing participant data. Randomization was stratified based on the current use of metformin, using a fixed block size of four and a permuted block design.

The duration of the lifestyle change intervention was 16 weeks. For adaptation to a healthy diet, all women consumed a TLC diet for two weeks prior to the start of the intervention. As a standard of care, all women were enrolled in an aerobic training program and received health counselling about PCOS (i.e., the value of lifestyle modifications in the management of the condition). Women were followed up at six and 12 months after the completion of the 16-week intervention to determine the long-term of effects of participating in the lifestyle intervention. The follow-up was completed to obtain the longitudinal effects of the lifestyle change program on the cardio-metabolic health outcomes of participants after the completion of the intervention when minimal instructions or counselling were provided by our multidisciplinary healthcare team on diet or physical activity.

All women were informed about both the dietary interventions before randomization. Participants were notified of diet allocation via email and thus were not blinded. Women who were randomized to the pulse-based diet group were aware the other group was to follow the TLC diet during the intervention, and vice versa. Although the participants were not blinded to the diet assignment, they were not aware of the hypothesis of the study. The allocation sequence was concealed from the dietitian who provided health counselling and those involved in assisting with exercise training and data entry. The participants and group allocations were coded, and the investigators collecting and analyzing data were also blinded to group assignment.

The pulse-based diet included soups, salads, and main course meals prepared with yellow split peas, green lentils, red split lentils, chickpeas, and pinto, black, and kidney beans. Two meals (i.e., lunch and dinner) were supplied daily for participants in the pulse-based diet group. Each meal contained approximately 90 g of split peas or 225 g of chickpeas or beans or 150 g of lentils (cooked weight). In the current study, the amount of dietary pulses in each meal was based on the amounts in the pulse-based diets that have been beneficial for reducing insulin, blood glucose, and lipid concentrations in prior investigations [23,27,28] which was higher than conventional healthy diets including the Dietary Approaches to Stop Hypertension eating plan [29] or the Mediterranean diet [30] that recommend some pulse intake. We examined whether a pulse-rich diet would exert similar health benefits in women with PCOS. According to the Eating Well with Canada’s Food Guide, the recommended serving of cooked legumes is 175 mL/day in the meat and alternative group [31] which is close to the amount supplied in our pulse-based diet. 

Prior to women receiving the two-week lead-in TLC diet and randomization, all women received an initial 1.5-h individualized dietary consultation session by a registered dietitian. During the session, all women received a booklet outlining TLC diet guidelines. The guidelines were developed by the NCEP Expert Panel on Detection, Evaluation, and Treatment of High Blood Cholesterol in Adults [24]. After randomization and before starting the intervention, a second dietitian who was not blinded to the randomization provided dietary consultation sessions to each of the pulse-based and TLC diet groups after all women followed the lead-in TLC diet for two weeks. Women who were randomized to the pulse-based diet were instructed to follow a pulse-rich diet in which two pulse meals were included per day (i.e., lunch and dinner) and TLC guidelines were to be followed for breakfast and snacks. Women who were randomized to the TLC diet were counselled to continue the TLC diet guideline and consume low-fat cuts of meat, poultry, and low fat or skim dairy as the main sources of protein and limit their pulse consumption. Energy restriction was not part of the protocol design [24]. As a standard of care, before randomization to the diet groups each participant received two sessions of education and counselling about PCOS and benefits of lifestyle change to manage PCOS for approximately four hours by a gynecologist, MSc, and PhD researchers who were knowledgeable about reproductive endocrinology and clinical nutrition. Each participant received standardized aerobic exercise training and was counselled to exercise for a minimum of 5 days/week for 45 min/day of low-impact aerobic activity at an intensity between 60–75% of age-predicted maximal heart rate (i.e., 220 minus age). Exercise consisted of brisk walking, training on elliptical, cycling, and rowing machines, depending on the participants’ preferences. Women who were randomized to the pulse-based diet group were counselled to follow the TLC diet recommendations for breakfast and snacks. During the intervention period, all women were monitored and supported for any issues related to the lifestyle change program and received monthly face-to-face individualized health counselling by a team of health professionals.

### 2.2. Participants

Recruitment was carried out by local newspaper advertisements, online bulletin posts, flyers available in physician offices, and placement of posters at the University of Saskatchewan and Royal University Hospital in Saskatoon, Canada. The diagnosis of PCOS was made according to the 2006 Androgen Excess and PCOS (AE-PCOS) Society criteria [32] and complied with the 2013 AE-PCOS Society recommendations for polycystic ovarian morphology (a threshold of ≥25 antral follicles measuring 2–9 mm in diameter) [33]. Barrier contraceptive methods and a negative pregnancy test were required. The diagnosis of MetS was made according to the 2009 International Diabetes Federation in collaboration with the American Heart Association/National Heart, Lung, and Blood Institute criteria [5].

### 2.3. Clinical Assessment

A standardized medical and physical examination was performed to obtain demographic, anthropometric, physiological, and gynecologic measures, familial history of diseases, and menstruation patterns as described previously [25,34]. Weight and height were measured using a mechanical weight scale (model 160KL; Health-O-Meter Inc., Bridgeview, IL, USA) and a portable stadiometer (Seca 208; Vogel and Halke, Hamburg, Germany). BMI was calculated using the formula: (body weight (kg)/(height squared) (m^2^). Waist circumference (WC) was measured following the World Health Organization (WHO) Waist Circumference Expert Consultation on Waist Circumference protocol [35]. Body composition was evaluated from whole-body scans by dual-energy X-ray absorptiometry (QDR Discovery Wi; Hologic Inc., Bedford, MD, USA). The coefficients of variation for total body fat mass, lean tissue, and trunk fat mass were 3.0%, 0.5%, and 5.0% respectively. Blood pressure was measured with a sphygmomanometer and a stethoscope (Littmann Master Classic, 3M Health Care, St. Paul, MN, USA).

### 2.4. Cardio-Metabolic Risk Profile Measurement

Endocrine and biochemical markers evaluated were total cholesterol (TC), LDL-C, high-density lipoprotein cholesterol (HDL-C), TC/HDL-C ratio, triglyceride (TG), highly sensitive C-reactive protein (hsCRP), and glycated hemoglobin (HbA1c); luteinizing hormone (LH), follicle stimulating hormone (FSH), total testosterone (TT), sex-hormone binding globulin (SHBG), dehydroepiandrosterone sulfate (DHEA-S), prolactin, thyroid stimulating hormone (TSH), and 17-hydroxyprogesterone (17-OHP) were assayed to exclude endocrinopathies that mimicked the phenotype or hormonal profile of PCOS. Insulin and glucose responses to a standard oral glucose tolerance test (OGTT) were examined before and after the 16-week intervention following the oral ingestion of a standard drink containing 75 g of glucose (Trutol 75; Thermo Scientific Inc., East Providence, RI, USA) as described elsewhere [25,36]. Fasting blood samples were collected between 8:00 and 9:00 a.m., following a 10–12 h overnight fast. Serial blood samples were taken pre-intervention, mid-intervention (nine weeks), post-intervention (16 weeks), and at long-term follow-up visits (six and 12 months post-intervention). All samples were analyzed by the Saskatoon Health Region Laboratories immediately or during the first week of the collection after freezing at −80 °C.

### 2.5. Biochemical Analyses

Plasma insulin (Alpco Diagnostics, Salem, NH, USA) was measured using a high-sensitivity enzyme-linked immunosorbent assay (ELISA), and plasma glucose (QuantiChrom, DIGL-100, BioAssay Systems, Hayward, CA, USA) by routine colorimetric technique, using commercial kits. Serum TT (by solid-phase, enzyme-labeled, competitive chemiluminescent immunoassay) was measured on Immulite 2000 Systems Analyzers (Siemens Healthcare Diagnostics Inc., Tarrytown, NY, USA) with Roche kits (Roche Diagnostics Ltd., Basel, Switzerland). The 17-OHP was measured by tandem mass spectrometry. The remaining compounds were analyzed using Roche Cobas Modular Analyzers and Roche kits (Roche Diagnostics Ltd., Basel, Switzerland), with HbA1c analyzed using the turbidimetric inhibition immunoassay; serum TG and cholesterol using the colourimetric assay; serum FSH and LH using the electrochemiluminescence immunoassay; serum SHBG using the solid-phase enzyme-labeled competitive chemiluminescent immunoassay; DHEA-S, TSH, and prolactin using the electrochemiluminescence immunoassay; and hsCRP using a particle-enhanced immunoturbidimetric assay. Homeostatic model assessment of IR (HOMA-IR) was calculated as previously described [37]. The intra- and inter-assay coefficients of variation were 5.1% to 10.3% and 6.7% to 16.6% for insulin respectively; <3% for glucose; 1.2% and 1.2% for HbA1c; 2.1% and 3% for cholesterol; 3.3% and 3.0% for HDL-C; 2.1% and 3.0% for LDL-C; 2.1% and 2.5% for TG; 3.5% and 0.8% for LH; 3.9% and 1.9% for 14 FSH; 6.8% and 5.4% for TT; 4.2% and 1.3% for SHBG; 14.3% and 2.3% for DHEA-S; 5.5% and 1.1% for prolactin; 1.4% and 2.9% for 13 TSH; 3.0% and 7.3% for 17-OHP; and 1.1% and 3.6% for hsCRP consistent with good assay performance.

### 2.6. Dietary and Physical Activity Assessment

Dietary intake was assessed using serial 24-h dietary recalls through a self-administered method. Dietary recalls were obtained at baseline and monthly during the 16-week intervention period. To standardize reporting portion sizes, we used a photo album that illustrated different portion sizes. All women were instructed about filling out the dietary recalls during dietary counselling sessions. Dietary intake data were analyzed using the ESHA Food Processor SQL Software (version 7.02, ESHA Research, Salem, OR, USA). GI was calculated according to the United Nations Food and Agriculture Organization/WHO [38] as described elsewhere [39]. The GI values were determined using the International Table of Glycemic Index Values and Glycemic Load (GL) [40]. For various foods reported in the recalls, the best matched GI was assigned by manually reviewing the table as previously used in several publications [41,42,43]. The GL of a serving of each food was calculated as (g of carbohydrate from food item × GI value of the food item)/100) [40,44]. The obtained dietary outcomes were compared with the National Institute of Health Dietary Reference Intakes [45].

Compliance with diet and physical activity were monitored using a daily diet and exercise log book as described previously [25]. Leisure time physical activity (i.e., physical activity outside the intervention) was evaluated using the Godin Leisure-Time Exercise Questionnaire [46]. The questionnaire was administered at baseline and monthly during the intervention. Total leisure time activity scores was expressed in arbitrary units and measured as described elsewhere [46].

### 2.7. Statistical Analysis

Statistical analyses were carried out according to intention-to-treat principles. Analyses were performed using SPSS for Windows (version 22.0; SPSS Inc., Chicago, IL, USA). Categorical variables were presented as numbers and percentages and continuous variables as mean ± SD, except in figures, where mean ± SEM was used for clarity. For between-group comparisons at baseline, Student’s *t*-tests were used for continuous variables with chi-squared analyses with the Pearson and Fisher’s Exact tests for categorical variables. To examine the differences in the outcome variables between women taking metformin compared with women not taking metformin, a three-factor analysis of variance (ANOVA), with between-group factors (pulse-based vs. TLC diet), and metformin (metformin users vs. non-users) and a within-subjects factor of time (baseline and post-intervention) was used. If there were no metformin-group by time interactions, metformin groups were combined, and a two factor ANOVA was used to compare responses between groups (the pulse-based vs. TLC diet groups) over all measurement time points (baseline, 16-week post-intervention, six- and 12-month follow up) to increase the statistical power of the ANOVA. The area under the two-h response versus time curves (AUC) for insulin and glucose was determined using the trapezium rule [47]. Incremental AUC, calculated as the increment in AUC over baseline concentrations, was used as summary measures of the postprandial insulin and glucose responses. Missing observations, defined as missing data points from women who completed the intervention, were assumed to be missing completely at random. Data collected at the nine-week time point (mid-intervention) were carried forward in place of any missing data at the post-intervention time point. To verify that mid-intervention data were an appropriate substitute for post-intervention, all analyses were also run using only participants who completed all testing time points (i.e., women who dropped out after mid-intervention were excluded from the secondary analysis). Different cohorts responded to each of the long-term follow-up examination time points; therefore, separate ANOVAs were used to assess participants who completed the six- and 12-months post-intervention time points. For each ANOVA result that was significantly different six- and 12-months post-intervention, pairwise comparisons were performed using a post hoc Bonferroni analysis to identify where the differences occurred. Changes in the mean fasting glucose from our previous pilot study (unpublished) on 28 women with PCOS were used to determine the required sample size for recruitment into the trial. Mean estimates with corresponding 95% confidence intervals were calculated. Results were considered significant at *p* ≤ 0.05.

Changes in glucose levels were −0.18 and 0.14 mmol/L for the pulse-based and TLC diet groups, respectively, with a population standard deviation of 0.46 and an effect size of −0.70. A sample size of 34 per group was determined to detect a significant difference in fasting glucose concentrations between groups at α = 0.05 with 80% power, assuming an anticipated dropout rate of 32%.

## 3. Results

**Figure 1:**
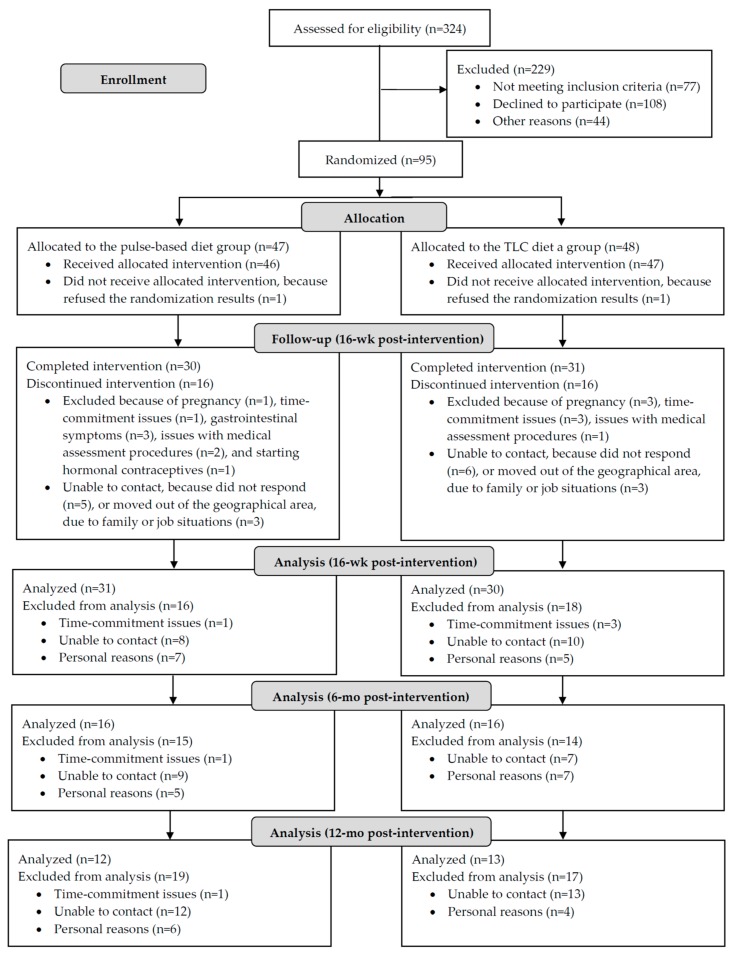
CONSORT flow diagram of the randomized controlled trial. In the “follow-up (16-week post-intervention)” section—“completed the intervention”—represented women who completed the 16-week lifestyle intervention;—“discontinued the intervention”—represented women who dropped out of the study before completing the 16-week lifestyle intervention. In the “analysis (16-week post-intervention)” section, the number of subjects that were analyzed in the pulse-based and TLC diet groups included women who completed the 16-week lifestyle intervention and women who dropped out of the study before completing the 16-week intervention period, but their last observation data which were collected at nine-weeks post-intervention were carried forward to 16-week time point according to the intention-to-treat principle.

**Figure 2:**
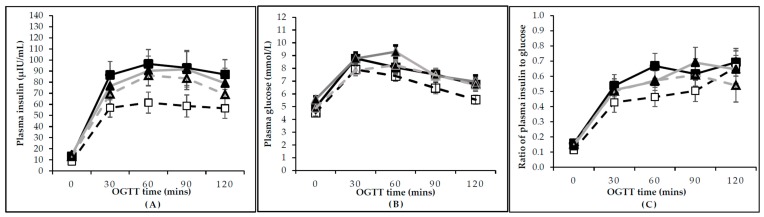
Plasma insulin and glucose responses to a standard 75-g OGTT before and after 16-week of intervention in the pulse-based diet and TLC diet groups. Solid dark lines represent women in the pulse-diet group at baseline (*n* = 30). Solid light lines represent women in the TLC diet group at baseline (*n* = 29). Dotted dark lines represent women in the pulse-diet group after the intervention (*n* = 30). Dotted light lines represent women in the TLC diet group after the intervention (*n* = 29). Insulin time course in response to OGTT (**A**); glucose time course in response to OGTT (**B**); ratio of plasma insulin to glucose time course in response to OGTT (**C**). Groups were comparable at baseline. Data are expressed as mean ± SEM. Abbreviations: OGTT, oral glucose tolerance test.

The flow-diagram of participants’ progress through the phases of the clinical trial according to the CONSORT statement, along with losses and exclusions, is displayed in Figure 1. A total of 324 women responded to the recruitment advertisement, and 95 were enrolled and randomized to the two diet groups. Following the CONSORT guidelines, baseline data for individuals that were randomized is presented in Table 1. Baseline characteristics, including the use of metformin (1000–1500 mg/day) were comparable in both diet groups (Table 1). Baseline characteristics of women who did not complete the 16-week intervention were not different from those who completed the intervention (*p* > 0.05; data not shown). The percentages of women who did not complete the 16-week intervention in the pulse-based (16/47; 34.0%) and TLC diet (18/48; 37.5%) groups was similar (Figure 1; *p* = 0.94). Thirty women in the pulse-based diet group and 31 in the TLC diet group completed the intervention. There were no missing observations for the anthropometric, body composition, physiologic, fasting insulin sensitivity, and hsCRP outcome measures presented in the results. Incomplete lipid profile (6/30 (20.0%) in the pulse-based and 6/31 (19.3%) in the TLC diet groups) and OGTT (2/31 (6.4%) in the TLC diet group) at the 16-week post-intervention time point were due to errors in collection, refusal by participants, or lack of personal time. Incomplete 24-h dietary recalls (7/30 (23.3%) in the pulse-based and 9/31 (29.0%) in the TLC diet groups) and physical activity records (3/30 (10.0%) in the pulse based and 1/31 (3.2%) in the TLC diet groups) were due to errors in collection, lack of personal time, or refusal by participants. Analyses of the six- and 12-month follow-up time points included 32 and 25 women, respectively: Of the women who completed the 16-week intervention 16/30 (53.3%) in the pulse based and 16/31 (51.6%) in the TLC diet group completed the six-month follow-up; 12/30 (40.0%) in the pulse-based and 13/31 (41.9%) in the TLC diet groups completed the 12-month follow-up (Figure 1). Baseline and post-intervention data for women who completed the 16-week intervention; six- and 12-months longitudinal follow-ups are found in Figure 2, Table 2 and Table 3; Table 4, Table 5, Table 6 and Table 7, respectively.

### 3.1. Metformin-Dietary Intervention Interactions

Of the women who completed the intervention 11 (36.7%) in the pulse-based and 13 (41.9%) in the TLC diet groups used metformin. There were no group by time by metformin interactions for any of the evaluated outcome measures (data not shown; *p* > 0.05).

### 3.2. Prevalence Rate of MetS

Changes in the prevalence of MetS over time in the pulse-based (from 36.7%; 11/30 to 30.0%; 9/30) and TLC (37.9%; 11/29 to 34.5%; 10/29) diet groups from the baseline were not different; no group by time interaction was observed (*p* = 0.78).

### 3.3. Anthropometric, Body Composition, and Blood Pressure Measures

Following the intervention, both pulse-based diet and TLC diet groups exhibited lower BMI (*p* = 0.01), WC (*p* = 0.02), and trunk fat mass (*p* < 0.0001) over time; no group by time interaction was observed. Similarly, there was a time main effect for total body fat mass (*p* < 0.01) and total % body fat (*p* < 0.01) with both decreasing across groups, without a group by time interaction (Table 2). Systolic blood pressure decreased following the intervention across groups (time main effect; *p* < 0.01), with no difference between groups. There was a group by time interaction for diastolic blood pressure (*p* = 0.05) with a greater decrease in the pulse-based diet compared to the TLC diet group (Table 2).

### 3.4. Insulin and Glucose Measurements and Responses to OGTT

Following the intervention, fasting plasma glucose (FPG; *p* < 0.01), fasting plasma insulin (*p* < 0.01), and HOMA-IR (*p* < 0.001) decreased over time, with no difference between groups (Table 2). Insulin and glucose responses to the OGGTs are shown in Figure 2. There was a group by time interaction (*p* = 0.05) for total insulin AUC with a greater decrease in the pulse-based diet group compared with the TLC diet group. Incremental insulin AUC (*p* = 0.03), total (*p* < 0.01), and incremental (*p* < 0.0001) glucose AUC decreased over time without a group by time interaction (Table 2). Of note, OGTT results were analyzed for 59 women (*n* = 30 in the pulse-based and *n* = 29 in the TLC diet groups) at baseline and post-intervention. There was an absence of mid-intervention OGTT data as an appropriate substitute for post-intervention. 

### 3.5. Lipid Profile

A group by time interaction was observed for TG (*p* = 0.04), HDL-C (*p* < 0.01), LDL-C (*p* = 0.05), and TC/HDL-C ratio (*p* < 0.001). The pulse-based diet group exhibited greater reductions in TG, LDL-C, TC/HDL-C ratio, and a greater increase in HDL-C concentrations when compared with the TLC diet group (Table 2). There was a time main effect (decrease) for TC (*p* < 0.01). To verify that mid-intervention data were an appropriate substitute for post-intervention, women who dropped out after mid-intervention were excluded from the analysis. Analyses of results using data only from participants who completed all testing time points showed a similar group by time interaction for TG (*p* < 0.01), HDL-C (*p* < 0.01), LDL-C (*p* = 0.05), and TC/HDL-C ratio (*p* < 0.0001). The pulse-based diet group showed greater reductions in TG, LDL-C, TC/HDL-C ratio, and a greater increase in HDL-C concentrations when compared with the TLC diet group (values not shown). Similarly, there was a time main effect (decrease) for TC (*p* < 0.01) for women who completed all testing time points.

### 3.6. Dietary Intake and Physical Activity

A mean diet adherence of 5.5 ± 0.4 and 5.3 ± 0.3 days/week was reported in the pulse-based and TLC diet groups, respectively (*p* = 0.12). During the intervention women in the pulse-based diet and TLC diet groups voluntarily reduced their average daily energy intake from baseline (time main effect; *p* < 0.0001). There were no differences for changes in the energy intake from baseline between the pulse-based and TLC diet groups (*p* = 0.97; Table 3). The pulse-based diet group (9.6% (47.6% to 57.2%)) had a greater increase in the percentage of carbohydrate intake during the intervention period when compared to the TLC diet group (3.0% (50.2% to 53.2%)) expressed as changes from baseline (*p* = 0.05). However, the pulse-based diet group (−16.7) exhibited a greater decrease in dietary GI levels from baseline when compared to the TLC diet group (−3.8; Table 3) due to increased consumption of low-GL foods (*p* < 0.01). The percentage of energy intake from dietary fats decreased from baseline in both pulse-based (−4.9% (34.2% to 29.3%)) and the TLC diet (−4.4% (34.5% to 30.1%)) groups over time during the intervention (*p* < 0.01) without a group by time interaction (*p* = 0.88). Changes in the percentage of energy intake from dietary proteins in the pulse-based (0.3% (16.8% to 16.5%)) and the TLC diet (1.8% (16.2% to 18.0%)) groups were comparable (*p* = 0.08), without a group by time interaction (*p* = 0.07). Overall, total carbohydrate, total fat, dietary cholesterol, saturated fat, trans fat, monounsaturated fat, polyunsaturated fat (PUFA), and total protein intakes decreased from baseline during the intervention (time main effect, *p* ≤ 0.05). The pulse-based diet group exhibited a greater decrease in cholesterol intake when compared to the TLC diet group (*p* < 0.001) and a higher intake of dietary fiber (*p* < 0.01). There was a time main effect (increase) for soluble fiber intake (*p* = 0.04). Vitamin B3 and B5 intakes decreased over time in both groups (*p* ≤ 0.05). Dietary intakes increased for folate (*p* = 0.001), vitamin K (*p* = 0.02), copper (*p* = 0.01), manganese (*p* = 0.001), magnesium (*p* = 0.04), and iron (*p* = 0.03) in the pulse-based diet group compared to the TLC diet group (Table 3). The pulse-based diet had a greater decrease in dietary sodium intake when compared to the TLC diet group (*p* = 0.05). The pulse-based diet exhibited a decrease in polyunsaturated omega-3 fatty acids intake when compared to the TLC diet group (*p* = 0.03, Table 3). There was a tendency toward decreased sugar intake (time main effect; *p* = 0.07) in the pulse-based (−15.9 g/day) and TLC diet (−11.0 g/day) groups from the baseline, without differences in the changes between the groups (*p* = 0.74). There were no changes in the dietary intake of other nutrients in response to intervention (data not shown).

The level of compliance with exercise was 53.1 ± 22.2 and 42.5 ± 8.6 min/day over five days/week for the pulse-based and TLC diet groups, respectively (*p* = 0.09). Leisure time physical activity scores increased during the intervention period, expressed as changes from baseline, in both the pulse based (32 ± 25 to 38 ± 29 arbitrary units) and the TLC (24 ± 20 to 34 ± 24 arbitrary units) diet groups (time main effect; *p* < 0.01); no differences were observed between groups (*p* = 0.53).

### 3.7. Adverse Events

Three participants who withdrew from the study reported four adverse events in the pulse-based diet group. The adverse events were upset stomach (*n* = 2), flatulence (*n* = 1), and bloating (*n* = 1). The adverse events were rated as mild to moderate in severity and were classified as “possibly” related to the intervention.

### 3.8. Long-Term Follow-Up: Clinical and Biochemical Measures Following Six and 12 Months Post-Intervention

Clinical and biochemical measures of women who participated in the six-month follow-up examination are presented in Table 4. Analyses of variance showed a group by time interaction for diastolic blood pressure (*p* = 0.01; Table 4). Results of the post hoc pairwise analysis showed, within the pulse-based diet group, diastolic blood pressure decreased at the 16-weeks versus the baseline (*p* = 0.03), but this decrease was not maintained six months post-intervention (*p* = 0.59). There was a group by time interaction for HDL-C levels six months post-intervention (*p* = 0.02; Table 4). Results of the pairwise post hoc comparisons using the Bonferroni corrections did not show any differences in the changes of the HDL-C levels within the intervention groups over the three time points (*p* ≥ 0.08). There was a group by time interaction for TC/HDL-C ratio (*p* = 0.02; Table 4). Results of the post hoc pairwise comparisons showed, within the pulse-based diet group, TC/HDL-C ratio decreased 16 weeks post-intervention (*p* < 0.01) and it was still lower at six months versus the baseline (*p* = 0.04).

Time main effects were evident for body weight (*p* < 0.00001), BMI (*p* < 0.0001), WC (*p* < 0.01), total body fat mass (*p* < 0.001), trunk fat mass (*p* = 0.01), total body fat % (*p* = 0.001) and systolic blood pressure (*p* = 0.01; Table 4). Results of the Bonferroni pairwise post hoc analyses expressed as changes from the 16-week post-intervention showed increased body weight (*p* < 0.0001) and BMI (*p* < 0.01) six months following the intervention. However, WC (*p* = 1.00), total body fat mass (*p* = 0.17), trunk fat mass (*p* = 1.00), total body fat % (*p* = 0.90), and systolic blood pressure (*p* = 1.00) levels remained unchanged over time between the 16-week and six-month time points. Analyses of variance showed a significant time main effect for fasting insulin levels (*p* < 0.01; Table 4). Results of the post hoc analyses showed increased levels of fasting insulin at six months when compared to the 16-week post-intervention (*p* = 0.01). Changes in TC concentrations were significant six months following the intervention (time main effect, *p* = 0.01; Table 4). Results of pairwise post hoc comparisons showed increased levels of TC at the six-month time point when compared to the 16-week post-intervention (*p* = 0.03). There was a time main effect for LDL-C levels (*p* < 0.01; Table 4). Both groups had a tendency toward increased levels of LDL-C six months following the intervention when compared to the 16-week time point based on the post hoc pairwise comparisons (*p* = 0.07).

Clinical and biochemical measures of women who participated in the 12-month follow-up examination are presented in Table 5. There was a group by time interaction for changes in the HDL-C levels (*p* = 0.02; Table 5). Results of the Bonferroni post hoc comparisons showed, within the pulse-based diet group, HDL-C concentrations increased from the baseline to the 16-week post-intervention (*p* = 0.02) and were still higher at the 12-month follow-up time point versus the baseline (*p* = 0.03). There was a group by time interaction for TC/HDL-C ratio (*p* < 0.01). Results of the post hoc analysis showed the TC/HDL-C ratio decreased from the baseline to the 16-week post-intervention within the pulse diet group (*p* < 0.01) and was lower 12 months after the completion of the intervention versus the baseline (*p* < 0.01).

Time main effects were evident for body weight (*p* = 0.01), BMI (*p* = 0.02), total body fat mass (*p* < 0.01), and total body fat % (*p* = 0.01) and systolic blood pressure (*p* = 0.02; Table 5). Results of the post hoc analyses showed no changes between the 16-week and the 12-month time points in the body weight (*p* = 0.17), BMI (*p* = 0.37), total body fat mass (*p* = 1.00), total body fat % (*p* = 1.00), and systolic blood pressure (*p* = 0.07) over time. There were time main effects for FPG (*p* = 0.04) and fasting insulin levels (*p* = 0.02, Table 5). Results of the post hoc analyses showed no changes in the levels of FPG 12 months after the intervention when compared to the 16-week post-intervention (*p* = 0.20). However, the levels of fasting insulin increased in both intervention groups at the 12-month time point when compared to the 16-week post-intervention (*p* = 0.05). There was a time main effect for TC levels 12 months post-intervention (*p* = 0.001; Table 5). Results of the pairwise post hoc analysis showed the levels of TC increased 12 months after the intervention when compared to the 16-week post-intervention levels (*p* < 0.01). There was a time main effect for LDL-C levels (*p* = 0.02; Table 5). Results of the post hoc analyses showed no changes in the LDL-C levels between the 16-week and 12-month time points (*p* = 0.70).

### 3.9. Long-Term Follow-Up: Dietary Intake and Physical Activity Following Six and 12 Months Post-Intervention

Dietary intakes of women who participated in the six- and 12-month follow-up examination are presented in Table 6 and Table 7. There was a group by time interaction for the dietary fiber intake six months post-intervention (*p* < 0.01; Table 6). Results of the post hoc pairwise comparisons showed, within the pulse-based diet group, consumption of dietary fiber increased during the intervention versus the baseline (*p* < 0.01); however, dietary fiber intake decreased in the pulse-based diet group six months post-intervention versus the baseline (*p* = 0.05). There was a time main effect for the dietary fiber intake at the 12-month follow-up time point (*p* = 0.02; Table 7). Results of the post hoc pairwise comparisons showed both the pulse-based and TLC diet groups consumed lower amounts of dietary fiber 12-month post-intervention (*p* = 0.02). Unlike the 12-month time point (*p* = 0.75; Table 7) there was a trend toward a time main effect for soluble fiber intake six months following the completion of the intervention (*p* = 0.06; Table 6). Post hoc comparisons between the intervention period and six months post-intervention showed that both the pulse-based and TLC diet groups had a tendency toward decreased intakes of soluble fiber (*p* = 0.06). There was a group by time interaction for manganese intake six months post-intervention (*p* = 0.03; Table 6). Results of the post hoc pairwise comparisons showed, within the pulse-based diet group, intakes of dietary manganese increased during the intervention from the baseline (*p* = 0.01), but decreased six months after the intervention versus the baseline (*p* < 0.01). There was a time main effect for manganese intake 12 months after the intervention (*p* = 0.001; Table 7). Results of the post hoc analysis showed a reduction in the dietary intake of manganese between the intervention and 12-month time points in both of the pulse-based and TLC diet groups (*p* < 0.001).

Significant time main effects were found for total energy intake six (*p* < 0.01) and 12 (*p* = 0.02) months following the completion of the intervention, without group by time interactions (Table 6 and Table 7). Results of the pairwise post hoc analyses showed both of the intervention groups maintained the reductions in total energy intake achieved during the intervention period six (Table 6) and 12 months post-intervention (Table 7). There were time main effects for the total fat intake six (*p* < 0.01) and 12 (*p* < 0.01) months following the intervention. Results of the pairwise post hoc analyses showed unlike the six-month time point (*p* = 0.31), the pulse-based and TLC diet groups showed a tendency toward increased intakes of total fat 12 months post-intervention, expressed as changes from the intervention period (*p* = 0.06). Unlike the 12-month time point (*p* = 0.13; Table 7), there was a time main effect for the protein intake six months following the completion of the intervention (*p* = 0.03; Table 6). Results of the post hoc pairwise comparisons showed increased intakes of protein at the six-month time point, expressed as changes from the intervention period (*p* = 0.03). There was a time main effect for the GI values of the food consumed by our participants six (*p* < 0.01; Table 6) and 12 (*p* < 0.01; Table 7) months following the completion of the intervention. Results of the post hoc pairwise comparisons showed the consumption of higher GI foods increased over time in the pulse-based and TLC diet groups six (*p* = 0.02) and 12 months (*p* < 0.01) post-intervention from the intervention period. Unlike the 12-month time point (*p* = 0.12; Table 7), there was a time main effect for magnesium intake six months following the intervention (*p* = 0.05; Table 6). Based on the results of the post hoc analysis, the pulse-based diet group exhibited a tendency toward decreased intakes of magnesium vs. the TLC diet group (*p* = 0.06) at the six-month time point, expressed as changes from the intervention period. There was a time main effect for sodium intake six months following the completion of the intervention (*p* < 0.01; Table 6), but not at the 12-month time point (*p* = 0.16; Table 7). Results of the post hoc analyses showed both the pulse-based and the TLC diet groups exhibited increased (*p* = 0.01) sodium intakes six months post-intervention from the intervention period. Unlike the six-month follow-up time point (*p* = 0.13; Table 6), there was a time main effect for the dietary potassium intake 12 months following the completion of the intervention (*p* = 0.04; Table 7). Results of the post hoc analysis showed both the diet groups decreased their intakes of potassium 12 months after the completion of the intervention from the intervention period (*p* = 0.03). There were no differences in the dietary intake of other nutrients between the intervention groups for any of the long-term follow-up time points (*p* > 0.05; data not shown).

Time main effects were evident for the scores of leisure time physical activity six (*p* = 0.001) and 12 (*p* = 0.02) months following the completion of the intervention. Results of the post hoc pairwise comparisons showed decreased scores of leisure time physical activity in both the pulse-based and TLC diet groups six months after the completion of the intervention when compared to the intervention period (−17 ± 23 vs. −7 ± 21 arbitrary units; *p* < 0.01). Both the pulse-based and TLC diet groups showed trends toward decreased scores of leisure-time physical activity over time 12 months after the completion of the intervention, expressed as changes from the intervention period (−16 ± 25 vs. −12 ± 18 arbitrary units; *p* = 0.08).

## 4. Discussion

The most significant finding from our PCOS study was that, without differences in the total energy intakes between the two diets, a low-GI pulse-based diet was likely more effective at decreasing total insulin AUC, levels of LDL-C, TG, TC/HDL-C ratio, diastolic blood pressure, and increasing the concentration of HDL-C than the TLC diet, in a multi-dimensional lifestyle change program, where all women participated in an exercise program, received education, and counselling about PCOS and the value of lifestyle modification. Improved lipid profiles were noteworthy as the control TLC diet is recommended to elicit LDL-C lowering effects in people at risk for CVD and DM2 [24]; the pulse-based diet was even more effective in the lowering effect on LDL-C. Improvements in both dietary groups were evident with glucoregulation, BMI, WC, trunk fat mass, % body fat, systolic blood pressure, and TC. While the benefits of the pulse-based diet were maintained for the levels of HDL-C and TC/HDL-C ratio, women in both groups tended to regain weight and revert to the baseline values for fasting insulin levels during the longitudinal follow-up at six and 12 months following the intervention.

Our observations are in agreement with previous studies in other populations that showed the positive effects of dietary pulses on risk indicators of MetS, CVD, and DM2 [20,22,23,49,50]. There was a greater reduction from baseline of TC by 4%, LDL-C by 8.2%, and TC/HDL-C ratio by 12.7% in the pulse diet group compared with the TLC diet group; by extrapolation, an estimated risk reduction of 8–12% in future major cardiovascular events may be realized [51,52]. Results of two meta-analyses of 26 RCTs (*n* = 1037) [20] and 10 RCTs (*n* = 268) [53] established evidence for a modest reduction in LDL-C (−0.17 to −0.21 mmol/L) and TC (−0.31 mmol/L), following the consumption of pulses over 3–16 weeks across age, BMI classes, and metabolic phenotypes. Hypocholesterolemic effects of pulses are multifactorial and can be attributed primarily to their high fiber content. In our study, the pulse-based diet group had a higher dietary fiber intake than the TLC diet group during the intervention (i.e., increase in fiber intake of ~10 vs. ~0 g/day, respectively), despite a comparable increase in the intake of soluble fiber. Pulses are a main source of dietary fiber and contain some soluble fiber. Soluble fiber, and in part, dietary fiber, modulate blood cholesterol by binding to bile acids in the intestine and increasing the excretion of bile acids and cholesterol through pre-established mechanisms [54,55,56]. However, increased bile acid excretion may be insufficient to account for the observed cholesterol reduction [57]. Increased intake of non-digestible dietary fiber, secondary to the consumption of a pulse-rich diet, can lead to the retention and improvement of gut microbiota composition, regulation of hepatic gluconeogenesis, lipogenesis, and lipid storage through mechanisms mediated by short-chain fatty acids, as signaling metabolites with prebiotic effects [56,58,59,60,61]. Our observations are consistent with other proposed changes in diet through which consuming pulse-rich foods can decrease hypercholesterolemia, including decreased intakes of dietary cholesterol and trans fats; higher intake of low-GI foods and subsequent increase in insulin sensitivity; higher intakes of protective nutrients such as minerals, folate, and antioxidant compounds, such as tannins, flavonoids, polyphenols, phytates, lectins, and saponins [19,50,55,62]. In the present study, women in the pulse-based diet group exhibited a higher improvement in the type of dietary fat intake, reflected by a greater decrease in dietary cholesterol and a tendency (i.e., *p* = 0.06) toward a greater decrease in trans fat intake when compared to the TLC diet group. Dietary consumption of trans fats and excessive dietary cholesterol intake have been shown to aggravate IR, dyslipidemia, and proinflammatory state [63,64]. Previous studies have shown the positive effects of PUFA intake on the metabolic health outcomes of PCOS including IR, abdominal adiposity, chronic-low inflammation, and dyslipidemia [65,66,67,68,69]. In our study, both groups had decreased intakes of PUFA and MUFA during the intervention, but total fat also decreased proportionately, which could explain the decreased intakes.

Following the intervention, the pulse-based diet group had a greater decrease in total insulin AUC during an OGTT when compared to the TLC diet group. An increase in insulin sensitivity in response to a pulse-based diet may be due to slow digestibility and, thus, lower glucose levels in the blood. Unlike the TLC diet group, women who were randomized to the pulse-based diet group achieved a low GI (≤45) during the intervention. A low GI diet has been recommended to reduce the risk of CVD and DM2 in the long-term [70]. Low GI of the pulse-rich foods has been attributed to complex carbohydrate profile, protein composition, protein-starch matrix, and anti-nutrient factors of pulses including phytates, saponins, lectins, and tannins [19,21,22,71]. Previous studies have shown poorer dietary choices in women with PCOS compared to non-PCOS controls, characterized by increased consumption of high-GI foods, saturated fats, and a low intake of dietary fiber, despite similarities in the overall energy and nutrient intakes [72,73]. The percentages of energy intake from macronutrients were within the acceptable macronutrient distribution range (AMDR) in our participants both at baseline and during the intervention. Our observations are consistent with previous reports on the proportion of energy intake from macronutrients in women with PCOS [72,73,74,75]. By contrast, at baseline, the average GI of the food consumed (56.6) was interpreted as moderate [76]. Our participants had a marginally low (23.6 g/day) intake of dietary fiber at baseline when compared to the adequate intake (AI) of 28 g/day [45]. Instead, our observations align with those of Barr et al. [77] and Moran et al. [78] on similarities between dietary GI and fiber intakes of women with PCOS and non-PCOS controls. It is crucial to acknowledge a reduction in the amount of carbohydrate intake *per se* may not be optimal or a practical strategy for all women with PCOS, including those with normal BMI, certain non-insulin resistant phenotypes, and women with obesity in long-term. The direct effect of pulses on glucoregulation, recommended dosage of pulses, and optimal duration of pulse consumption needed to prevent insulin resistance, impaired glucose tolerance and DM2 is less clear [79,80,81].

In addition to improving lipid and insulin profiles, the pulse-based diet group exhibited a 4.4% greater reduction in diastolic blood pressure when compared to the TLC diet group. A reduction of this magnitude for diastolic blood pressure is estimated to reduce the risk of myocardial infarction by 9–13% [51]. Jayalath et al. performed a meta-analysis on eight isocaloric trials (*n* = 554 participants) and showed pulse consumption (~162 g/day) over ten weeks reduced mean arterial blood pressure by −0.75 mmHg in middle-aged subjects [49]. A diet rich in pulses shares many nutritional characteristics with the Dietary Approaches to Stop Hypertension eating plan [29] and the Mediterranean diet [30] that are endorsed for the prevention and treatment of hypertension. Pulses can confer blood pressure-lowering effects by increasing the dietary intakes of plant proteins, potassium, magnesium, folate, low-GI foods, dietary fiber, as well as decreasing intake of dietary sodium. High sodium intake is associated with increased glucocorticoid synthesis, IR, hyperglyceridemia, and lower adiponectin levels [82,83]. The sodium intake of our participants exceeded the American Heart Association recommendations (≤2.4 g/day) at baseline, consistent with the sodium intake of American women with PCOS [73]. The mean sodium intake of the pulse-based diet group was still higher than the adequate intake (AI; 1.5 g/day) during the intervention; however, the decrease in the sodium intake of the pulse-based diet group was greater compared to the TLC diet group. The observed decrease in the diastolic blood pressure in the pulse-based diet group may also be attributed to the regulation of the renin-angiotensin-aldosterone system, secondary to decreased IR through pre-established mechanisms [19,84,85,86,87].

During the intervention, we observed changes in the micronutrient intakes of groups, including achieving the recommended intakes of folate, magnesium, and iron in the pulse-based diet group and decreased iron intake in the TLC diet group relative to the recommended dietary allowance (RDA) for adult women [45]. The intake of folate was enhanced with a pulse-based diet. In addition to positive effects on endothelial function and modulating blood pressure [88,89], adequate folate intake has been associated with a decreased risk of endometrial carcinoma [90,91]. Women with PCOS are at two to six-fold increased risk of endometrial carcinoma [92]. However, it is difficult to derive a conclusion about the positive effect of the pulse-based diet on micronutrients adequacy based on the RDA values *per se*. There is a significant potential to overestimate the dietary requirement of women who do not meet the recommended RDA cut-offs because the values have been defined to meet the recommendations of 97.5% of the North American population [45,93].

Lifestyle management research in PCOS has been focused primarily upon short-term energy restriction designed to achieve weight loss. In overweight and obese women with PCOS, weight loss has been considered effective to improve PCOS health outcomes [15,17,94]. However, weight loss through energy restriction often has been less attainable or sustainable [95]. Innovative PCOS-specific diets have been largely overlooked as a means to achieve persistent, long-term lifestyle change. The optimal dietary recommendations for obese and non-obese women with PCOS have been debated. Our results align with the findings of Marsh et al. [96] who found positive effects on insulin sensitivity with an *ad libitum* low-GI diet when compared with a macronutrient- and fiber-matched healthy diet. Increased insulin sensitivity was attributed to the consumption of low-GI foods and a modest weight loss (4–5% of baseline body weight). It should be noted that the low-GI diet of the Marsh et al. study had very few pulse-based foods, and in contrast to our study, no improvement in lipid profiles was observed. Similarly, Barr et al. reported increased insulin sensitivity following an isocaloric low-GI non-randomized intervention in women with PCOS, without changes in LDL-C and TG levels independent from weight change [97]. While our study goals were not focused on weight loss, our subjects achieved a 5% weight loss across both diet groups. The observed weight loss can be attributed to a voluntary decrease in energy intake following education about lifestyle modification and an increase in physical activity. A 5–10% weight loss is considered clinically significant and has been associated with a substantial improvement in the metabolic profile and risk factors for CVD and DM2 [98,99]. Apart from inducing satiety and weight loss, several factors can explain the beneficial effects of a pulse-based diet on improving glycemic response to OGTT including high fiber content, low-GI, significant anti-oxidant content, and a favorable micronutrient and macronutrient composition [19,21,22].

In addition to the benefits of dietary modification, aerobic exercise training, education, and health counselling might have contributed to improved cardio-metabolic health profile of our participants. Physical activity has been shown to improve the risk factors of CVD and DM2 in women with PCOS [100,101,102]. Education and health counselling might have improved our participants’ lifestyle behaviors, motivation, self-monitoring, and adherence to the intervention protocol as informed by data from general populations [103]. 

The participant attrition rate was high (33.7%) in both groups in the current study. However, high attrition rates (27–49%) have been reported in other RCTs focused on lifestyle changes in women with PCOS with a progressive increase in dropouts corresponding with the duration of intervention [18,96,104,105]. Difficulties in sustaining a new lifestyle program, the time-consuming nature of the intervention, and competing family, work, and school obligations were among the reasons that contributed to the attrition rate of our participants. During the intervention period of the trial, we found regular communication for assessments, provision of education, encouragement, and feedback by a multidisciplinary healthcare team improved our participants’ adherence to new lifestyle strategies specific to women with PCOS and likely decreased the rate of attrition.

Longitudinal assessment of the study showed that women experienced the greatest improvements in anthropometric, body composition, blood pressure, insulin sensitivity, and lipid outcomes 16-weeks post-intervention when compared to six- or 12-month follow-up time points. While some of the post-intervention improvements (e.g., HDL-C and TC/HDL-C ratio) in the pulse-based diet group were maintained, women tended to revert to the pre-intervention levels for cardio-metabolic profile in long-term, reflected by increased levels of fasting insulin and body weight. Women tended to regain weight in the long-term consistent with previous studies [106,107]. These observations may be attributed to decreased levels of physical activity and less favorable dietary intakes in both groups. In the present study, the longitudinal assessment was performed without the continual support of a multidisciplinary healthcare team to ensure adherence to lifestyle behaviors. The duration of our intervention might not have been adequate to sustain and maintain the newly adopted healthy lifestyle practices in our participants in the long-term. Successful and sustainable adherence to healthy lifestyle change programs has been challenging with less favorable and mixed results in the context of PCOS [6,108]. Long-term therapeutic strategies are crucial to prevent or delay the occurrence of comorbidities and improve the overall prognosis associated with PCOS [6]. Our observations highlight the importance of regular follow-up visits with a multidisciplinary team to monitor, support, motivate, and encourage women with PCOS to increase their adherence to the newly adopted healthy lifestyle practices.

Limitations of our study include a high attrition rate, yet one that is not uncommon in PCOS intervention studies. The inability to detect FPG differences between groups post-intervention may be due to the study being underpowered. Due to time and financial constraints, we were unable to contrast the isolated effects of each of the pulse-based and TLC diets in women with PCOS against a non-PCOS cohort of women in reproductive age. We used serial 24-h dietary recalls as the most accurate and least biased instrument of reporting dietary intake. However, our participants’ dietary recalls may have a tendency toward random or systematic error, underreporting, and reactivity [109,110]. We observed a poor response rate to the dietary recalls. Thereby, our results may be skewed toward responders. The benefits of a pulse-based diet on cardio-metabolic health outcomes over the TLC diet may be interpreted with caution. 

Strengths of the study included a well-defined and diagnosed PCOS population; adopting a multi-dimensional approach comprised of dietary, exercise, education, and health counselling strategies to evaluate changes in cardio-metabolic health indicators in response to treatment in women with PCOS; measurement of biochemical parameters at defined timelines to ensure uniformity of sampling; and long-term follow-up of participants, which is a challenging and neglected component in PCOS research.

## 5. Conclusions

In conclusion, in a diet intervention without prescribed energy restriction, where aerobic exercise was part of a healthy lifestyle program, and health counselling was provided, a pulse-based diet may be more effective than the TLC diet for improving insulin response to an OGTT, levels of TG, LDL-C, HDL-C, TC/HDL-C ratio, and diastolic blood pressure, which can be translated to improved cardio-metabolic and DM2 risk profiles in women with PCOS. Our results support the position that lifestyle modifications are crucial in the management of PCOS and add another dimension to the existing evidence concerning the positive effects of pulse consumption on cardio-metabolic risk profile in women with PCOS. Future longitudinal research should address the compliance, attrition rate, and barriers to lifestyle modifications in PCOS. Attempts should be made to develop effective strategies for more successful engagement of women with PCOS with lifestyle modification that is sustainable.

## Figures and Tables

**Table 1:** Baseline characteristics of women with PCOS.

	Pulse-Based Diet Group	TLC Diet Group	*p* Value *
Age (year)	27.0 ± 4.6	26.9 ± 4.4	0.91
Ethnicity (*n* (%))			0.69
Caucasian	35 (74.5)	36 (75.0)	
Asian	8 (17.0)	10 (20.8)	
Indigenous	1 (2.1)	0	
African	1 (2.1)	0	
Latin American	2 (4.3)	2 (4.2)	
Metformin Tx (*n* (%))	18 (38.3)	20 (41.7)	0.45
Anthropometrics and body composition measures			
Weight (kg)	87.4 ± 23.9	92.2 ± 24.5	0.33
BMI (kg/m^2^)	32.5 ± 8.4	33.3 ± 9.0	0.65
WC (cm)	102.4 ± 19.8	103.5 ± 20.6	0.78
Total body fat mass (kg)	36.1 ± 13.0	39.6 ± 16.7	0.26
Trunk fat mass (kg)	16.4 ± 6.4	18.4 ± 8.1	0.21
Total body fat (%)	41.3 ± 7.2	42.3 ± 8.5	0.54
Total body lean mass (kg)	46.4 ± 7.3	48.8 ± 9.5	0.18
Physiologic measures			
SBP (mmHg)	115 ± 8	117 ± 11	0.33
DBP (mmHg)	76 ± 7	77 ± 9	0.81
Pulse rate (beats/min)	74 ± 14	74 ± 14	0.82
Total energy intake (kcal/day)	2165 ± 774	2128 ± 720	0.82
Leisure physical activity score ^†^ (arbitrary units)	28 ± 25	20 ± 19	0.08
Family history of DM2 (*n* (%))	34 (77.3)	32 (76.2)	0.55
Family history of CVD and/or HTN (*n* (%))	32 (72.7)	34 (81.0)	0.26
Insulin sensitivity measures			
FPG (mmol/L)	5.2 ± 1.4	5.3 ± 1.3	0.68
Fasting insulin (µIU/mL)	13.0 ± 10.0	15.6 ± 12.2	0.25
HbA1c (%)	5.2 ± 0.4	5.3 ± 0.5	0.27
HOMA-IR index	3.0 ± 2.3	4.0 ± 4.0	0.16
Fasting insulin/glucose ratio	0.2 ± 0.1	0.2 ± 0.1	0.55
Lipid profile			
TC (mmol/L)	4.7 ± 1.0	4.5 ± 0.7	0.38
TG (mmol/L)	1.4 ± 0.8	1.3 ± 0.7	0.81
HDL-C (mmol/L)	1.3 ± 0.3	1.3 ± 0.4	0.80
LDL-C (mmol/L)	2.8 ± 0.9	2.6 ± 0.7	0.31
TC/HDL-C ratio	3.8 ± 1.2	3.7 ± 1.3	0.84
Prevalence of MetS ^‡^ (*n* (%))	18 (38.3)	16 (33.3)	0.67
Presence of hirsutism ^⸹^ (*n* (%))	32 (68.1)	35 (72.9)	0.39
Endocrine parameters			
LH/FSH ratio	2.2 ± 1.1	2.6 ± 1.7	0.32
TT (nmol/L)	1.5 ± 0.5	1.7 ± 1.1	0.41
SHBG (nmoL/L)	32.0 ± 18.7	36.5 ± 25.8	0.60
DHEA-S (µmol/L)	6.3 ± 2.2	5.8 ± 2.2	0.29
Prolactin (µg/L)	12.1 ± 3.6	11.8 ± 3.4	0.73
TSH (mIU/L)	2.2 ± 0.9	2.3 ± 0.9	0.75
17-OHP (nmol/L)	2.3 ± 1.2	2.1 ± 0.8	0.38
hsCRP (mg/L)	4.0 ± 3.8	5.5 ± 6.6	0.18

Abbreviations: PCOS, polycystic ovary syndrome; TLC, Therapeutic Lifestyle Changes; Tx, therapy; BMI, body mass index; WC, waist circumference; SBP, systolic blood pressure; DBP, diastolic blood pressure; DM2, type 2 diabetes; CVD, cardiovascular disease; HTN, hypertension; FPG, fasting plasma glucose; HbA1c, glycated hemoglobin; HOMA-IR, homeostatic model assessment of insulin resistance; TC, total cholesterol; TG, triglyceride; HDL-C, high-density lipoprotein cholesterol; LDL-C, low-density lipoprotein cholesterol; TC/HDL-C, ratio of total cholesterol/high-density lipoprotein cholesterol; LH/FSH, ratio of luteinizing hormone to follicle stimulating hormone; TT, total testosterone; SHBG, sex–hormone binding globulin; DHEA-S, dehydroepiandrosterone sulfate; TSH, thyroid stimulating hormone; 17-OHP, 17-hydroxyprogesterone; hsCRP, highly sensitive C–reactive protein. Data are expressed as mean ± SD except indicated otherwise. Numbers in each group for baseline characteristics of all women who were enrolled in the study were as follows: Pulse-based diet group = 47; TLC diet group = 48. * Student *t*-test and chi-squared test were used for comparisons of means and proportions between groups. ^†^ Determined using the Godin Leisure-Time Exercise Questionnaire [46]. ^‡^ Determined according to the 2009 International Diabetes Federation in collaboration with the American Heart Association/National Heart, Lung, and Blood Institute criteria [5]. ^⸹^ Determined using the Ferriman–Gallwey Index, adjusted for ethnicity [48].

**Table 2:** Cardio-metabolic outcomes at baseline and after 16 weeks of intervention.

	Pulse-Based Diet Group			TLC Diet Group			*p* Value *	
	Baseline	16 Weeks	Change	Baseline	16 Weeks	Change	Time	Group × Time
Anthropometric and body composition measures								
Weight (kg)	89.9 ± 27.0	84.4 ± 26.8	−5.5 ± 4.5	93.3 ± 25.4	88.4 ± 23.0	−4.9 ± 15.8	<0.01	0.62
BMI (kg/m^2^)	33.3 ± 9.0	32.0 ± 9.0	−1.3 ± 1.4	34.0 ± 9.8	32.2 ± 8.6	−1.8 ± 6.1	0.01	0.62
WC (cm)	103.9 ± 19.8	99.5 ± 18.0	−4.4 ± 11.2	103.5 ± 20.2	101.8 ± 19.3	−1.7 ± 7.6	0.02	0.30
Total body fat mass (kg)	36.3 ± 13.5	34.6 ± 13.8	−1.7 ± 2.4	40.5 ± 15.0	37.5 ± 15.3	−3.0 ± 7.5	<0.01	0.41
Trunk fat mass (kg)	16.0 ± 6.8	14.9 ± 6.4	−1.1 ± 2.0	19.3 ± 8.3	17.3 ± 8.1	−2 ± 3.9	<0.0001	0.25
Total body fat (%)	41.1 ± 7.2	40.1 ± 7.8	−1.0 ± 2.0	41.4 ± 8.7	40.4 ± 8.5	−1.0 ± 2.4	<0.01	0.96
Total body lean mass (kg)	47.5 ± 8.1	46.8 ± 8.1	−0.7 ± 2.2	49.5 ± 9.1	49.7 ± 9.3	0.2 ± 14.1	0.22	0.09
Physiologic measures								
SBP (mmHg)	116 ± 7	113 ± 10	−3 ± 8	118 ± 10	113 ± 10	−5 ± 8	<0.001	0.45
DBP (mmHg)	77 ± 7	74 ± 8	−3 ± 7	77 ± 9	77 ± 10	0 ± 7	0.03	0.05
Pulse rate (beats/min)	74 ± 14	74 ± 14	0 ± 10	73 ± 12	73 ± 14	0 ± 12	0.89	0.89
Insulin sensitivity measures								
FPG (mmol/L)	5.0 ± 1.5	4.6 ± 1.3	−0.4 ± 1.7	5.6 ± 1.4	4.8 ± 1.6	−0.8 ± 1.5	<0.01	0.38
Fasting insulin (µIU/mL)	14.0 ± 11.4	10.0 ± 7.7	−4.0 ± 9.7	15.7 ± 12.4	12.7 ± 10.3	−3.0 ± 6.8	<0.01	0.60
HbA1c (%)	5.3 ± 0.4	5.2 ± 0.4	−0.1 ± 0.3	5.3 ± 0.5	5.3 ± 0.4	0.0 ± 0.3	0.18	0.71
HOMA-IR index	3.1 ± 2.5	2.1 ± 1.9	−1.0 ± 2.1	4.2 ± 4.4	2.9 ± 3.6	−1.3 ± 2.1	<0.001	0.66
Fasting insulin/glucose ratio	0.2 ± 0.1	0.1 ± 0.1	−0.0 ± 0.1	0.2 ± 0.1	0.1 ± 0.1	−0.1 ± 0.1	0.21	0.17
Total insulin AUC (µIU/mL × min)	326.9 ± 266.5	205.9 ± 106.7	−121.0 ± 229.9	307.2 ± 181.7	279.8 ± 176.7	−27.4 ± 110.2	<0.01	0.05
Incremental insulin AUC (µIU/mL × min)	49.8 ± 45.7	32.5 ± 22.4	−17.3 ± 47.2	45.6 ± 26.1	41.1 ± 29.3	−4.5 ± 22.1	0.03	0.19
Total glucose AUC (mmol/L × min)	32.3 ± 9.6	26.8 ± 5.3	−5.5 ± 10.4	33.7 ± 5.8	29.7 ± 7.1	−4.0 ± 6.3	<0.01	0.77
Incremental glucose AUC (mmol/L × min)	6.7 ± 1.9	6.2 ± 1.4	−0.5 ± 2.3	7.2 ± 1.5	6.4 ± 1.5	−0.8 ± 1.5	<0.0001	0.51
Lipid profile								
TC (mmol/L)	5.0 ± 1.0	4.6 ± 0.8	−0.4 ± 0.5	4.4 ± 0.8	4.3 ± 0.8	−0.1 ± 0.5	<0.01	0.12
TG (mmol/L)	1.5 ± 0.8	1.3 ± 0.7	−0.2 ± 0.6	1.3 ± 0.7	1.3 ± 0.8	0 ± 0.5	0.36	0.04
HDL-C (mmol/L)	1.3 ± 0.3	1.4 ± 0.3	0.1 ± 0.2	1.3 ± 0.4	1.2 ± 0.3	−0.1 ± 0.2	0.64	<0.01
LDL-C (mmol/L)	2.9 ± 0.4	2.7 ± 0.8	−0.2 ± 0.4	2.6 ± 0.7	2.5 ± 0.6	−0.1 ± 0.4	<0.01	0.05
TC/HDL-C	4.0 ± 1.2	3.6 ± 1.1	−0.4 ± 0.4	3.7 ± 1.3	3.8 ± 1.3	0.1 ± 0.4	0.01	<0.001
hsCRP (mg/L)	4.2 ± 3.8	3.9 ± 4.8	−0.3 ± 3.4	5.0 ± 6.4	5.0 ± 8.2	0.0 ± 4.2	0.78	0.72

Abbreviations: TLC, Therapeutic Lifestyle Changes; BMI, body mass index; WC, waist circumference; SBP, systolic blood pressure; DBP, diastolic blood pressure; FPG, fasting plasma glucose; HbA1c, glycated hemoglobin; HOMA-IR, homeostatic model assessment of insulin resistance; AUC, Area under the curve; TC, total cholesterol; TG, triglyceride; HDL-C, high-density lipoprotein cholesterol; LDL-C, low-density lipoprotein cholesterol; TC/HDL-C, ratio of total cholesterol/high-density lipoprotein cholesterol; hsCRP, highly sensitive C–reactive protein. Data are expressed as mean ± SD. Numbers in each group were as follows: Pulse-based diet group = 31 (30 for insulin sensitivity data); TLC diet group = 30 (29 for insulin sensitivity data). * Repeated measures ANOVA. Group main effects were not significant (*p* > 0.05).

**Table 3:** Dietary intakes at baseline and during the intervention.

	Pulse-Based Diet Group			TLC Diet Group			*p* Value *	
	Baseline	16 Weeks	Change	Baseline	16 Weeks	Change	Time	Group × Time
Total energy intake (kcal/day)	2273 ± 724	1707 ± 27	−566 ± 667	2211 ± 536	1654 ± 406	−557 ± 696	<0.0001	0.97
Carbohydrate intake (g/day)	272.3 ± 100.4	243.5 ± 64.1	−28.8 ± 93.4	281.5 ± 95.1	221.2 ± 71.8	−60.3 ± 106.8	<0.01	0.30
Fat intake (g/day)	88.0 ± 42.0	56.7 ± 24.8	−31.3 ± 43.6	84.6 ± 27.5	55.3 ± 19.8	−29.3 ± 37.0	<0.0001	0.87
Protein intake (g/day)	92.0 ± 29.3	69.1 ± 15.7	−22.9 ± 24.9	87.3 ± 25.7	75.3 ± 25.7	−12.0 ± 34.6	<0.001	0.24
Dietary fiber (g/day)	22.8 ± 7.8	33.3 ± 8.2	10.5 ± 10.2	24.5 ± 14.6	24.5 ± 9.5	0.0 ± 14.4	<0.01	<0.01
Soluble fiber (g/day)	1.2 ± 1.3	1.5 ± 1.5	0.3 ± 1.3	1.3 ± 1.4	1.9 ± 1.7	0.6 ± 1.5	0.04	0.45
Cholesterol intake (mg/day)	357 ± 185	128 ± 114	−229 ± 20.6	285 ± 155	288 ± 165	3 ± 196	<0.01	<0.001
Saturated fat (g/day)	27.9 ± 12.2	14.0 ± 5.4	−13.9 ± 13.1	24.7 ± 15.7	17.4 ± 9.9	−7.3 ± 18.8	<0.0001	0.18
Trans fat (g/day)	1.3 ± 1.9	0.4 ± 0.5	−0.9 ± 2.0	0.4 ± 0.4	0.3 ± 0.4	−0.1 ± 0.5	0.04	0.06
Monounsaturated fat (g/day)	25.2 ± 14.2	19.0 ± 5.9	−6.2 ± 13.5	22.1 ± 12.5	16.9 ± 8.4	−5.2 ± 12.0	<0.01	0.78
Polyunsaturated fat (g/day)	13.4 ± 12.0	9.9 ± 3.3	−3.5 ± 11.2	11.1 ± 6.5	8.8 ± 3.3	−2.3 ± 7.0	0.05	0.68
Glycemic index ^†^	55 ± 7	39 ± 7	−16 ± 10	54 ± 8	50 ± 8	−4 ± 8	<0.0001	<0.01
Glycemic load ^‡^	142 ± 60	96 ± 25	−46 ± 57	163 ± 58	101 ± 26	−62 ± 49	<0.0001	0.43
Vitamin B3 (mg/day)	29 ± 24	21 ± 18	−8 ± 17	26 ± 23	21 ± 10	−5 ± 25	0.04	0.64
Vitamin B5 (mg/day)	9 ± 14	8 ± 9	−1 ± 7	8 ± 12	5 ± 5	−3 ± 8	0.05	0.47
Folate (µg/day)	380 ± 205	639 ± 266	259 ± 287	344 ± 260	295 ± 159	−49 ± 271	0.02	0.001
Vitamin K (µg/day)	76 ± 41	147 ± 83	71 ± 85	91 ± 122	102 ± 107	11 ± 80	<0.01	0.02
Copper (µg/day)	1542 ± 1127	2068 ± 918	526 ± 960	1412 ± 1169	1060 ± 439	−352 ± 1193	0.60	0.01
Manganese (mg/day)	3 ± 1	5 ± 2	2 ± 2	3 ± 3	3.0 ± 1.1	−0 ± 3	0.03	0.001
Magnesium (mg/day)	284 ± 120	357 ± 116	73 ± 115	279 ± 145	271 ± 96	−8 ± 141	0.01	0.04
Iron (mg/day)	17 ± 8	19 ± 5	2 ± 7	22 ± 21	13 ± 4	−9 ± 22	0.16	0.03
Sodium (g/day)	3.7 ± 2.2	1.7 ± 0.5	−2.0 ± 2.2	3.1 ± 1.6	2.4 ± 1.5	−0.7 ± 2.1	<0.001	0.05
Omega-3 polyunsaturated fatty acids (g/day)	0.6 ± 0.6	0.3 ± 0.5	−0.3 ± 0.6	0.5 ± 0.7	0.7 ± 0.9	0.2 ± 0.8	0.99	0.03

Data are expressed as mean ± SD. Numbers for dietary intake in each group were as follows: Pulse-based diet group = 23; TLC diet group = 22. * Repeated measures ANOVA; ^†^ Glycemic index scale (glucose = 100); ^‡^ Glycemic index multiplied by the mean total available carbohydrate intake per day divided by 100. Group main effects were not significant (*p* > 0.05).

**Table 4:** Cardio-metabolic outcomes of women who completed the six-month follow up phase of the study at baseline, 16 weeks, and six months post-intervention.

	Pulse-Based Diet Group			TLC Diet Group			*p* Value *	
	Baseline	16 Weeks	Six Months	Baseline	16-Weeks	Six Months	Time	Group × Time
Anthropometric and body composition measures								
Weight (kg)	81.6 ± 13.5	78.0 ± 12.6 ^a^	79.6 ± 13.4 ^b^	95.8 ± 21.1	92.0 ± 20.5 ^a^	94.8 ± 18.1 ^b^	<0.00001	0.43
BMI (kg/m^2^)	30.4 ± 6.2	29.0 ± 5.6 ^a^	31.2 ± 7.2 ^b^	35.1 ± 9.1	33.9 ± 8.9 ^a^	34.7 ± 8.8 ^b^	<0.0001	0.30
WC (cm)	98.0 ± 13.7	94.6 ± 13.6 ^a^	94.9 ± 14.2	108.2 ± 20.6	105.5 ± 17.7 ^a^	105.5 ± 17.1	<0.01	0.95
Total body fat mass (kg)	32.3 ± 10.7	29.8 ± 10.1 ^a^	30.3 ± 9.9 ^a^	42.7 ± 14.3	40.3 ± 14.3 ^a^	41.5 ± 14.9 ^a^	<0.001	0.67
Trunk fat mass (kg)	15.0 ± 5.9	14.0 ± 5.7 ^a^	14.8 ± 5.5	20.3 ± 8.3	19.1 ± 7.9 ^a^	18.4 ± 6.7	0.01	0.39
Total body fat (%)	38.9 ± 7.8	37.3 ± 8.1 ^a^	37.4 ± 7.6 ^a^	43.8 ± 8.2	42.3 ± 8.0 ^a^	43.0 ± 8.1 ^a^	0.001	0.52
Total body lean mass (kg)	46.5 ± 4.4	45.7 ± 4.7	46.7 ± 4.9	51.0 ± 8.4	51.1 ± 8.5	51.4 ± 8.6	0.18	0.42
Physiologic measures								
SBP (mmHg)	115 ± 8	112 ± 11 ^a^	112 ± 9	121 ± 10	115 ± 9 ^a^	118 ± 11	0.01	0.49
DBP (mmHg)	75 ± 7	71 ± 7 ^a^	74 ± 8	81 ± 7	81 ± 5	77 ± 8	0.11	0.01
Pulse rate (beats/min)	73 ± 14	70 ± 13.9	69 ± 15	73 ± 16	74 ± 13	77 ± 10	0.78	0.27
Insulin sensitivity measures								
FPG (mmol/L)	5.3 ± 1.7	4.6 ± 1.6	4.9 ± 0.2	5.5 ± 1.5	4.6 ± 1.8	5.3 ± 0.9	0.08	0.86
Fasting insulin (µIU/mL)	13.5 ± 12.8	9.8 ± 8.7 ^a^	13.3 ± 11.2 ^b^	14.6.1 ± 3.6	10.4 ± 11.9 ^a^	16.7 ± 9.1 ^b^	<0.01	0.83
Lipid profile								
TC (mmol/L)	4.7 ± 1.1	4.4 ± 0.8 ^a^	4.6 ± 0.8 ^b^	4.4 ± 0.8	4.2 ± 0.8 ^a^	4.4 ± 0.7 ^b^	<0.01	0.90
TG (mmol/L)	1.4 ± 0.9	1.3 ± 0.8	1.3 ± 0.7	1.2 ± 0.6	1.2 ± 0.6	1.3 ± 0.6	0.68	0.86
HDL-C (mmol/L)	1.2 ± 0.3	1.3 ± 0.4	1.3 ± 0.4	1.3 ± 0.4	1.2 ± 0.3	1.2 ± 0.2	0.98	0.02
LDL-C (mmol/L)	2.8 ± 1.0	2.4 ± 0.8	2.6 ± 0.8	2.5 ± 0.7	2.4 ± 0.6	2.6 ± 0.5	<0.01	0.35
TC/HDL-C	4.0 ± 1.5	3.6 ± 1.4 ^a^	3.6 ± 1.2 ^a^	3.5 ± 0.8	3.5 ± 1.0	3.6 ± 0.8	<0.01	0.02
hsCRP (mg/L)	3.2 ± 3.4	2.4 ± 2.8	2.9 ± 4.1	5.2 ± 7.3	6.3 ± 10.8	5.6 ± 7.4	0.99	0.22

Abbreviations: TLC, Therapeutic Lifestyle Changes; BMI, body mass index; WC, waist circumference; SBP, systolic blood pressure; DBP, diastolic blood pressure; FPG, fasting plasma glucose; TC, total cholesterol; TG, triglyceride; HDL-C, high–density lipoprotein cholesterol; LDL-C, low-density lipoprotein cholesterol; TC/HDL-C, ratio of total cholesterol/high-density lipoprotein cholesterol; hsCRP, highly sensitive C–reactive protein. Data are expressed as mean ± SD. Numbers in each group were as follows: Pulse-based diet group = 16; TLC diet group = 16. * Repeated measure ANOVA. ^a^ Significantly different from the baseline (*p* ≤ 0.05) as determined by the repeated measures ANOVA and pairwise comparisons with Bonferroni corrections. ^b^ Significantly different from the 16-week post-intervention (*p* ≤ 0.05) as determined by the repeated measures ANOVA and pairwise comparisons with Bonferroni corrections. Group main effects were not significant (*p* > 0.05).

**Table 5:** Cardio-metabolic outcomes of women who completed the 12-month follow up phase of the study at baseline, 16 weeks, and 12 months post-intervention.

	Pulse-Based Diet Group			TLC Diet Group			*p* Value *	
	Baseline	16 Weeks	12 Months	Baseline	16 Weeks	12 Months	Time	Group × Time
Anthropometric and body composition measures								
Weight (kg)	84.0 ± 14.6	78.9 ± 11.7 ^a^	80.4 ± 14.0	96.1 ± 24.6	92.8 ± 24.9 ^a^	95.9 ± 27.4	0.01	0.53
BMI (kg/m^2^)	31.6 ± 6.0	30.0 ± 5.5 ^a^	30.7 ± 6.8	35.5 ± 10.9	34.5 ± 10.6 ^a^	35.3 ± 11.2	0.02	0.74
WC (cm)	98.7 ± 11.6	94.4 ± 9.3	93.6 ± 11.4	107.6 ± 24.6	104.3 ± 20.9	105.5 ± 21.7	0.13	0.66
Total body fat mass (kg)	32.2 ± 11.1	29.4 ± 9.2 ^a^	29.8 ± 9.9	42.1 ± 16.2	40.9 ± 16.5 ^a^	41.3 ± 16.9	<0.01	0.34
Trunk fat mass (kg)	14.6 ± 5.3	13.6 ± 5.3	13.5 ± 8.0	19.0 ± 9.7	18.3 ± 9.4	17.8 ± 8.0	0.52	0.70
Total body fat (%)	39.4 ± 8.0	37.7 ± 7.2 ^a^	38.2 ± 7.6	44.6 ± 8.4	43.9 ± 8.0 ^a^	43.6 ± 7.9	0.01	0.45
Total body lean mass (kg)	45.6 ± 3.5	45.4 ± 3.9	45.2 ± 4.3	48.7 ± 10.2	48.4 ± 1.0	49.4 ± 1.0	0.49	0.50
Physiologic measures								
SBP (mmHg)	112 ± 8	109 ± 11 ^a^	114 ± 8	121 ± 11	112 ± 9 ^a^	117 ± 11	0.02	0.17
DBP (mmHg)	76 ± 6	73 ± 7	76 ± 8	79 ± 8	78 ± 6	78 ± 6	0.12	0.64
Pulse rate (beats/min)	76 ± 16	71 ± 13	70 ± 10	73 ± 17	74 ± 14	77 ± 18	0.85	0.50
Insulin sensitivity measures								
FPG (mmol/L)	5.2 ± 1.1	4.3 ± 0.8 ^a^	4.9 ± 0.6	5.5 ± 1.4	4.7 ± 1.9 ^a^	5.3 ± 0.5	0.04	0.98
Fasting insulin (µIU/mL)	16.3 ± 15.3	11.4 ± 11.2 ^a^	14.1 ± 9.6 ^b^	18.0 ± 17.3	11.9 ± 12.6 ^a^	16.5 ± 10.0 ^b^	0.02	0.90
Lipid profile								
TC (mmol/L)	4.8 ± 1.1	4.5 ± 0.9 ^a^	5.0 ± 1.1 ^b^	4.8 ± 0.5	4.5 ± 0.5 ^a^	4.7 ± 0.7 ^b^	0.001	0.43
TG (mmol/L)	1.5 ± 0.8	1.2 ± 0.5	1.4 ± 0.6	1.2 ± 0.6	1.2 ± 0.5	1.4 ± 0.6	0.41	0.38
HDL-C (mmol/L)	1.1 ± 0.2	1.3 ± 0.4 ^a^	1.3 ± 0.3 ^a^	1.4 ± 0.4	1.3 ± 0.2	1.4 ± 0.4	0.22	0.02
LDL-C (mmol/L)	3.0 ± 1.1	2.6 ± 0.8 ^a^	2.9 ± 1.0	2.7 ± 0.5	2.6 ± 0.4 ^a^	2.5 ± 0.7	0.02	0.15
TC/HDL-C	4.4 ± 1.3	3.8 ± 1.2 ^a^	3.7 ± 0.9 ^a^	3.6 ± 0.8	3.5 ± 0.7	3.6 ± 0.8	<0.01	<0.01
hsCRP (mg/L)	4.5 ± 4.0	5.0 ± 7.6	5.8 ± 4.4	5.9 ± 7.7	7.1 ± 11.4	8.0 ± 14.4	0.48	0.95

Abbreviations: TLC, Therapeutic Lifestyle Changes; BMI, body mass index; WC, waist circumference; SBP, systolic blood pressure; DBP, diastolic blood pressure; FPG, fasting plasma glucose; TC, total cholesterol; TG, triglyceride; HDL-C, high–density lipoprotein cholesterol; LDL-C, low-density lipoprotein cholesterol; TC/HDL-C, ratio of total cholesterol/high-density lipoprotein cholesterol; hsCRP, highly sensitive C–reactive protein. Data are expressed as mean ± SD. Numbers in each group were as follows: Pulse-based diet group = 12; TLC diet group = 13. * Repeated measure ANOVA. ^a^ Significantly different from the baseline (*p* ≤ 0.05) as determined by the repeated measures ANOVA and pairwise comparisons with Bonferroni corrections. ^b^ Significantly different from the 16-week post-intervention (*p* ≤ 0.05) as determined by the repeated measures ANOVA and pairwise comparisons with Bonferroni corrections. Group main effects were not significant (*p* > 0.05).

**Table 6:** Dietary intakes of women who completed the six-month follow up phase of the study at baseline, 16 weeks, and six months post-intervention.

	Pulse-Based Diet Group			TLC Diet Group			*p* Value *	
	Baseline	16 Weeks	Six Months	Baseline	16-Weeks	Six Months	Time	Group × Time
Total energy intake (kcal/day)	2020 ± 625	1634 ± 195 ^a^	1762 ± 288	2213 ± 525	1545 ± 297 ^a^	1687 ± 386	<0.01	0.63
Fat intake (g/day)	77.9 ± 21.4	49.9 ± 7.1 ^a^	66.8 ± 25.5	78.8 ± 27.8	58.6 ± 19.1 ^a^	62.3 ± 8.9	<0.01	0.56
Protein intake (g/day)	94.3 ± 34.7	66.6 ± 10.3	88.8 ± 22.7 ^b^	85.7 ± 26.9	71.1 ± 21.7	82.5 ± 17.4 ^b^	0.03	0.62
Dietary fiber (g/day)	24.0 ± 8.0	32.3 ± 7.3 ^a^	18.7 ± 5.0 ^a, b^	20.3 ± 8.4	20.3 ± 7.7	21.8 ± 8.0	<0.01	<0.01
Soluble fiber (g/day)	1.0 ± 1.4	1.3 ± 1.3	1.1 ± 1.0	0.9 ± 1.0	1.3 ± 1.1	0.6 ± 0.7	0.06	0.45
Glycemic index ^†^	53 ± 5	38 ± 5 ^a^	55 ± 5 ^b^	55 ± 5	49 ± 6 ^a^	61 ± 2 ^b^	<0.01	0.41
Magnesium (mg/day)	278 ± 113	349 ± 102	249 ± 74	318 ± 173	241 ± 92	218 ± 63	0.05	0.14
Manganese (mg/day)	3 ± 2	5 ± 2 ^a^	3 ± 1 ^b^	4 ± 4	3 ± 1	3 ± 1	<0.01	0.03
Sodium (g/day)	3.8 ± 2.5	1.7 ± 0.6 ^a^	3.1 ± 1.0 ^b^	3.0 ± 0.7	2.1 ± 1.1 ^a^	3.0 ± 1.4 ^b^	<0.01	0.11
Potassium (mg/day)	2916 ± 1051	3062 ± 874	2508 ± 472	2517 ± 966	1978 ± 647	1862 ± 731	0.13	0.38

Data are expressed as mean ± SD. Numbers for dietary intake in each group were as follows: Pulse-based diet group = 16; TLC diet group = 16. * Repeated measures ANOVA; ^†^ Glycemic index scale (glucose = 100). ^a^ Significantly different from the baseline (*p* ≤ 0.05) as determined by the repeated measures ANOVA and pairwise comparisons with Bonferroni corrections. ^b^ Significantly different from the 16-week post-intervention (*p* ≤ 0.05) as determined by the repeated measures ANOVA and pairwise comparisons with Bonferroni corrections. Group main effects were not significant (*p* > 0.05).

**Table 7:** Dietary intakes of women who completed the 12-month follow up phase of the study at baseline, 16 weeks, and 12 months post-intervention.

	Pulse-Based Diet Group			TLC Diet Group			*p* Value *	
	Baseline	16 Weeks	12 Months	Baseline	16-Weeks	12 Months	Time	Group × Time
Total energy intake (kcal/day)	2126 ± 686	1583 ± 306 ^a^	1735 ± 358	2205 ± 673	1577 ± 361 ^a^	1749 ± 324	0.02	0.97
Fat intake (g/day)	74.5 ± 33.5	48.9 ± 7.5 ^a^	67.5 ± 25.9	73.9 ± 31.9	44.7 ± 13.6 ^a^	58.5 ± 20.3	<0.01	0.67
Protein intake (g/day)	91.1 ± 25.5	73.3 ± 15.3	87.3 ± 18.5	81.8 ± 31.2	79.7 ± 27.4	82.6 ± 27.9	0.13	0.09
Dietary fiber (g/day)	20.8 ± 9.3	31.9 ± 7.4 ^a^	21.8 ± 6.5 ^b^	19.0 ± 9.4	25.0 ± 7.9 ^a^	20.0 ± 9.0 ^b^	0.02	0.53
Soluble fiber (g/day)	1.2 ± 1.2	1.6 ± 1.9	1.6 ± 1.3	1.3 ± 1.4	1.6 ± 1.4	1.0 ± 1.6	0.75	0.27
Glycemic index ^†^	54 ± 6	38 ± 4 ^a^	46 ± 8 ^b^	54 ± 5	50 ± 3 ^a^	56 ± 8 ^b^	<0.01	0.29
Magnesium (mg/day)	262 ± 125	349 ± 95	259 ± 167	243 ± 176	307 ± 74	242 ± 100	0.12	0.93
Manganese (mg/day)	3 ± 2	5 ± 1	2 ± 1 ^b^	3 ± 3	3 ± 1	2 ± 1^b^	0.001	0.09
Sodium (g/day)	3.6 ± 2.7	1.7 ± 0.3	2.5 ± 1.8	2.8 ± 1.1	2.3 ± 1.1	2.5 ± 1.6	0.16	0.40
Potassium (mg/day)	2848 ± 1384	3021 ± 915	1683 ± 1001 ^b^	2215 ± 1132	2383 ± 929	1646 ± 942 ^b^	0.04	0.68

Data are expressed as mean ± SD. Numbers for dietary intake in each group were as follows: Pulse-based diet group = 12; TLC diet group = 13. * Repeated measures ANOVA; ^†^ Glycemic index scale (glucose = 100); ^a^ Significantly different from the baseline (*p* ≤ 0.05) as determined by the repeated measures ANOVA and pairwise comparisons with Bonferroni corrections. ^b^ Significantly different from the 16-week post-intervention (*p* ≤ 0.05) as determined by the repeated measures ANOVA and pairwise comparisons with Bonferroni corrections. Group main effects were not significant (*p* > 0.05).

## Supplementary Materials

The following are available online at http://www.mdpi.com/2072-6643/10/10/1387/s1, CONSORT 2010 checklist of information to include when reporting a randomised trial.

## References

[1] March W.A., Moore V.M., Willson K.J., Phillips D.I.W., Norman R.J., Davies M.J. (2010). The prevalence of polycystic ovary syndrome in a community sample assessed under contrasting diagnostic criteria. Hum. Reprod..

[2] Carmina E., Lobo R.A. (1999). Polycystic ovary syndrome (PCOS): Arguably the most common endocrinopathy is associated with significant morbidity in women. J. Clin. Endocrinol. Metab..

[3] Diamanti-Kandarakis E., Dunaif A. (2012). Insulin resistance and the polycystic ovary syndrome revisited: An update on mechanisms and implications. Endocr. Rev..

[4] Wild R.A., Carmina E., Diamanti-Kandarakis E., Dokras A., Escobar-Morreale H.F., Futterweit W., Lobo R., Norman R.J., Talbott E., Dumesic D.A. (2010). Assessment of cardiovascular risk and prevention of cardiovascular disease in women with the polycystic ovary syndrome: A consensus statement by the Androgen Excess and Polycystic Ovary Syndrome (AE-PCOS) Society. J. Clin. Endocrinol. Metab..

[5] Alberti K.G.M.M., Eckel R.H., Grundy S.M., Zimmet P.Z., Cleeman J.I., Donato K.A., Fruchart J.C., James W.P.T., Loria C.M., Smith S.C. (2009). Harmonizing the metabolic syndrome: A joint interim statement of the International Diabetes Federation Task Force on Epidemiology and Prevention; National Heart, Lung, and Blood Institute; American Heart Association; World Heart Federation; International Atherosclerosis Society; and International Association for the Study of Obesity. Circulation.

[6] Marsh K., Brand-Miller J. (2005). The optimal diet for women with polycystic ovary syndrome?. Br. J. Nutr..

[7] Cooney L.G., Milman L.W., Hantsoo L., Kornfield S., Sammel M.D., Allison K.C., Epperson C.N., Dokras A. (2018). Cognitive-behavioral therapy improves weight loss and quality of life in women with polycystic ovary syndrome: A pilot randomized clinical trial. Fertil. Steril..

[8] Dokras A., Stener-Victorin E., Yildiz B.O., Li R., Ottey S., Shah D., Epperson N., Teede H. (2018). Androgen Excess-Polycystic Ovary Syndrome Society: Position statement on depression, anxiety, quality of life, and eating disorders in polycystic ovary syndrome. Fertil. Steril..

[9] Moran L.J., Hutchison S.K., Norman R.J., Teede H.J. (2011). Lifestyle changes in women with polycystic ovary syndrome. Cochrane Database Syst. Rev..

[10] Teede H.J., Misso M.L., Costello M.F., Dokras A., Laven J., Moran L., Piltonen T., Norman R.J., International P.N. (2018). Recommendations from the international evidence-based guideline for the assessment and management of polycystic ovary syndrome. Hum. Reprod..

[11] Moran L.J., Ko H., Misso M., Marsh K., Noakes M., Talbot M., Frearson M., Thondan M., Stepto N., Teede H.J. (2013). Dietary composition in the treatment of polycystic ovary syndrome: A systematic review to inform evidence-based guidelines. J. Acad. Nutr. Diet..

[12] Asemi Z., Samimi M., Tabassi Z., Shakeri H., Sabihi S.S., Esmaillzadeh A. (2014). Effects of DASH diet on lipid profiles and biomarkers of oxidative stress in overweight and obese women with polycystic ovary syndrome: A randomized clinical trial. Nutrition.

[13] Asemi Z., Esmaillzadeh A. (2015). DASH diet, insulin resistance, and serum hs-CRP in polycystic ovary syndrome: A randomized controlled clinical trial. Horm. Metab. Res..

[14] Toscani M.K., Mario F.M., Radavelli-Bagatini S., Wiltgen D., Matos M.C., Spritzer P.M. (2011). Effect of high-protein or normal-protein diet on weight loss, body composition, hormone, and metabolic profile in southern Brazilian women with polycystic ovary syndrome: A randomized study. Gynecol. Endocrinol..

[15] Stamets K., Taylor D.S., Kunselman A., Demers L.M., Pelkman C.L., Legro R.S. (2004). A randomized trial of the effects of two types of short-term hypocaloric diets on weight loss in women with polycystic ovary syndrome. Fertil. Steril..

[16] Thomson R.L., Buckley J.D., Noakes M., Clifton P.M., Norman R.J., Brinkworth G.D. (2008). The effect of a hypocaloric diet with and without exercise training on body composition, cardiometabolic risk profile, and reproductive function in overweight and obese women with polycystic ovary syndrome. J. Clin. Endocrinol. Metab..

[17] Mehrabani H.H., Salehpour S., Amiri Z., Farahani S.J., Meyer B.J., Tahbaz F. (2012). Beneficial effects of a high-protein, low-glycemic-load hypocaloric diet in overweight and obese women with polycystic ovary syndrome: A randomized controlled intervention study. J. Am. Coll. Nutr..

[18] Turner-McGrievy G.M., Davidson C.R., Wingard E.E., Billings D.L. (2014). Low glycemic index vegan or low-calorie weight loss diets for women with polycystic ovary syndrome: A randomized controlled feasibility study. Nutr. Res..

[19] Mudryj A.N., Yu N., Aukema H.M. (2014). Nutritional and health benefits of pulses. Appl. Physiol. Nutr. Metab..

[20] Ha V., Sievenpiper J.L., de Souza R.J., Jayalath V.H., Mirrahimi A., Agarwal A., Chiavaroli L., Mejia S.B., Sacks F.M., Di Buono M. (2014). Effect of dietary pulse intake on established therapeutic lipid targets for cardiovascular risk reduction: A systematic review and meta-analysis of randomized controlled trials. Can. Med. Assoc. J. CMAJ.

[21] McCrory M.A., Hamaker B.R., Lovejoy J.C., Eichelsdoerfer P.E. (2010). Pulse consumption, satiety, and weight management. Adv. Nutr..

[22] Sievenpiper J.L., Kendall C.W.C., Esfahani A., Wong J.M.W., Carleton A.J., Jiang H.Y., Bazinet R.P., Vidgen E., Jenkins D.J.A. (2009). Effect of non-oil-seed pulses on glycaemic control: A systematic review and meta-analysis of randomised controlled experimental trials in people with and without diabetes. Diabetologia.

[23] Abeysekara S., Chilibeck P.D., Vatanparast H., Zello G.A. (2012). A pulse-based diet is effective for reducing total and LDL-cholesterol in older adults. Br. J. Nutr..

[24] National Cholesterol Education Program (NCEP) Expert Panel on Detection, Evaluation, and Treatment of High Blood Cholesterol in Adults (Adult Treatment Panel III) (2002). Third report of the National Cholesterol Education Program (NCEP) Expert Panel on Detection, Evaluation, and Treatment of High Blood Cholesterol in adults (Adult Treatment Panel III) final report. Circulation.

[25] McBreairty L.E., Chilibeck P.D., Chizen D.R., Pierson R.A., Tumback L., Sherar L.B., Zello G.A. (2017). The role of a pulse-based diet on infertility measures and metabolic syndrome risk: Protocol of a randomized clinical trial in women with polycystic ovary syndrome. BMC Nutr..

[26] Canadian Institutes of Health Research, Natural Science and Engineering Research Council, Social Sciences and Humanities Research Council of Canada Tri-Council Policy Statement: Ethical Conduct for Research Involving Humans. http://www.ethics.gc.ca/pdf/eng/tcps2/TCPS_2_FINAL_Web.pdf.

[27] Schäfer G., Schenk U., Ritzel U., Ramadori G., Leonhardt U. (2003). Comparison of the effects of dried peas with those of potatoes in mixed meals on postprandial glucose and insulin concentrations in patients with type 2 diabetes. Am. J. Clin. Nutr..

[28] Shutler S.M., Bircher G.M., Tredger J.A., Morgan L.M., Walker A.F., Low A.G. (1989). The effect of daily baked bean (*Phaseolus vulgaris*) consumption on the plasma lipid levels of young, normo-cholesterolaemic men. Br. J. Nutr..

[29] Sacks F.M., Svetkey L.P., Vollmer W.M., Appel L.J., Bray G.A., Harsha D., Obarzanek E., Conlin P.R., Miller E.R., Simons-Morton D.G. (2001). Effects on blood pressure of reduced dietary sodium and the Dietary Approaches to Stop Hypertension (DASH) diet. N. Engl. J. Med..

[30] Willett W.C., Sacks F., Trichopoulou A., Drescher G., Ferro-Luzzi A., Helsing E., Trichopoulos D. (1995). Mediterranean diet pyramid: A cultural model for healthy eating. Am. J. Clin. Nutr..

[31] Health Canada Eating Well with Canada’s Food Guide. http://www.hc-sc.gc.ca/fn-an/alt_formats/hpfb-dgpsa/pdf/food-guide-aliment/print_eatwell_bienmang-eng.pdf.

[32] Azziz R., Carmina E., Dewailly D., Diamanti-Kandarakis E., Escobar-Morreale H.F., Futterweit W., Janssen O.E., Legro R.S., Norman R.J., Taylor A.E. (2006). Criteria for defining polycystic ovary syndrome as a predominantly hyperandrogenic syndrome: An Androgen Excess Society guideline. J. Clin. Endocrinol. Metab..

[33] Dewailly D., Lujan M.E., Carmina E., Cedars M.I., Laven J., Norman R.J., Escobar-Morreale H.F. (2014). Definition and significance of polycystic ovarian morphology: A task force report from the Androgen Excess and Polycystic Ovary Syndrome Society. Hum. Reprod. Update.

[34] Clark N.M., Podolski A.J., Brooks E.D., Chizen D.R., Pierson R.A., Lehotay D.C., Lujan M.E. (2014). Prevalence of polycystic ovary syndrome phenotypes using updated criteria for polycystic ovarian morphology: An assessment of over 100 consecutive women self-reporting features of polycystic ovary syndrome. Reprod. Sci..

[35] World Health Organization (2011). Waist Circumference and Waist-Hip Ratio: Report of a WHO Expert Consultation. Geneva, Switzerland, 8–11 December 2008.

[36] Goldenberg R., Punthakee Z. (2013). Definition, classification and diagnosis of diabetes, prediabetes and metabolic syndrome. Can. J. Diabetes.

[37] Matthews D.R., Hosker J.P., Rudenski A.S., Naylor B.A., Treacher D.F., Turner R.C. (1985). Homeostasis model assessment: Insulin resistance and beta-cell function from fasting plasma glucose and insulin concentrations in man. Diabetologia.

[38] World Health Organization (2000). Preventing and Managing the Global Epidemic.

[39] Graff S.K., Mário F.M., Alves B.C., Spritzer P.M. (2013). Dietary glycemic index is associated with less favorable anthropometric and metabolic profiles in polycystic ovary syndrome women with different phenotypes. Fertil. Steril..

[40] Atkinson F.S., Foster-Powell K., Brand-Miller J.C. (2008). International Tables of Glycemic Index and Glycemic Load Values: 2008. Diabetes Care.

[41] Egan N., Read A., Riley P., Atiomo W. (2011). Evaluating compliance to a low glycaemic index (GI) diet in women with polycystic ovary syndrome (PCOS). BMC Res. Notes.

[42] Jenkins D.J., Kendall C.W., Augustin L.S., Franceschi S., Hamidi M., Marchie A., Jenkins A.L., Axelsen M. (2002). Glycemic index: Overview of implications in health and disease. Am. J. Clin. Nutr..

[43] Beulens J.W., de Bruijne L.M., Stolk R.P., Peeters P.H., Bots M.L., Grobbee D.E., van der Schouw Y.T. (2007). High dietary glycemic load and glycemic index increase risk of cardiovascular disease among middle-aged women: A population-based follow-up study. J. Am. Coll. Cardiol..

[44] Salmerón J., Manson J.E., Stampfer M.J., Colditz G.A., Wing A.L., Willett W.C. (1997). Dietary fiber, glycemic load, and risk of non-insulin-dependent diabetes mellitus in women. JAMA.

[45] Nutrient Recommendations: Dietary Reference Intakes (DRI). https://ods.od.nih.gov/Health_Information/Dietary_Reference_Intakes.aspx.

[46] Godin G., Shephard R. (1985). A simple method to assess exercise behaviour in the community. Can. J. Appl. Sport Sci..

[47] Tai M.M. (1994). A mathematical model for the determination of total area under glucose tolerance and other metabolic curves. Diabetes Care.

[48] Yildiz B.O., Bolour S., Woods K., Moore A., Azziz R. (2010). Visually scoring hirsutism. Hum. Reprod. Update.

[49] Jayalath V.H., De Souza R.J., Sievenpiper J.L., Ha V., Chiavaroli L., Mirrahimi A., Di Buono M., Bernstein A.M., Leiter L.A., Kris-Etherton P.M. (2014). Effect of dietary pulses on blood pressure: A systematic review and meta-analysis of controlled feeding trials. Am. J. Hypertens..

[50] Anderson J.W., Major A.W. (2002). Pulses and lipaemia, short- and long-term effect: Potential in the prevention of cardiovascular disease. Br. J. Nutr..

[51] Manson J.E., Tosteson H., Ridker P.M., Satterfield S., Hebert P., O’Connor G.T., Buring J.E., Hennekens C.H. (1992). The primary prevention of myocardial infarction. N. Engl. J. Med..

[52] Mihaylova B., Emberson J., Blackwell L., Keech A., Simes J., Barnes E.H., Voysey M., Gray A., Collins R., Cholesterol Treatment Trialists’ (CTT) Collaborators (2012). The effects of lowering LDL cholesterol with statin therapy in people at low risk of vascular disease: Meta-analysis of individual data from 27 randomised trials. Lancet.

[53] Bazzano L.A., Thompson A.M., Tees M.T., Nguyen C.H., Winham D.M. (2011). Non-soy legume consumption lowers cholesterol levels: A meta-analysis of randomized controlled trials. Nutr. Metab. Cardiovasc. Dis..

[54] Galisteo M., Duarte J., Zarzuelo A. (2008). Effects of dietary fibers on disturbances clustered in the metabolic syndrome. J. Nutr. Biochem..

[55] Brown L., Rosner B., Willett W.W., Sacks F.M. (1999). Cholesterol-lowering effects of dietary fiber: A meta-analysis. Am. J. Clin. Nutr..

[56] Kishimoto Y., Wakabayashi S., Takeda H. (1995). Hypocholesterolemic effect of dietary fiber: Relation to intestinal fermentation and bile acid excretion. J. Nutr. Sci. Vitaminol. (Tokyo).

[57] Pilch S.M., Center for Food Safety and Applied Nutrition, Federation of American Societies for Experimental Biology, Life Sciences Research Office (Contributors) (1987). Physiological Effects and Health Consequences of Dietary Fiber.

[58] Koh A., De Vadder F., Kovatcheva-Datchary P., Bäckhed F. (2016). From dietary fiber to host physiology: Short-chain fatty acids as key bacterial metabolites. Cell.

[59] Van Bennekum A.M., Nguyen D.V., Schulthess G., Hauser H., Phillips M.C. (2005). Mechanisms of cholesterol-lowering effects of dietary insoluble fibres: Relationships with intestinal and hepatic cholesterol parameters. Br. J. Nutr..

[60] Chibbar R.N., Ambigaipalan P., Hoover R. (2010). Review: Molecular diversity in pulse seed starch and complex carbohydrates and its role in human nutrition and health. Cereal Chem..

[61] Schneeman B.O. (1987). Dietary fiber and gastrointestinal function. Nutr. Rev..

[62] Ludwig D.S. (2002). The glycemic index: Physiological mechanisms relating to obesity, diabetes, and cardiovascular disease. JAMA.

[63] Mozaffarian D., Pischon T., Hankinson S.E., Rifai N., Joshipura K., Willett W.C., Rimm E.B. (2004). Dietary intake of trans fatty acids and systemic inflammation in women. Am. J. Clin. Nutr..

[64] Lefevre M., Lovejoy J.C., Smith S.R., DeLany J.P., Champagne C., Most M.M., Denkins Y., de Jonge L., Rood J., Bray G.A. (2005). Comparison of the acute response to meals enriched with cis-or trans-fatty acids on glucose and lipids in overweight individuals with differing FABP2 genotypes. Metabolism.

[65] Diamanti-Kandarakis E., Alexandraki K., Piperi C., Protogerou A., Katsikis I., Paterakis T., Lekakis J., Panidis D. (2006). Inflammatory and endothelial markers in women with polycystic ovary syndrome. Eur. J. Clin. Investig..

[66] Velazquez E., Bellabarba G.A., Mendoza S., Sánchez L. (2000). Postprandial triglyceride response in patients with polycystic ovary syndrome: Relationship with waist-to-hip ratio and insulin. Fertil. Steril..

[67] Bahcecı M., Aydemır M., Tuzcu A. (2007). Effects of oral fat and glucose tolerance test on serum lipid profile, apolipoprotein, and CRP concentration, and insulin resistance in patients with polycystic ovary syndrome. Fertil. Steril..

[68] Kirchengast S., Huber J. (2001). Body composition characteristics and body fat distribution in lean women with polycystic ovary syndrome. Hum. Reprod..

[69] Kasim-Karakas S.E., Almario R.U., Gregory L., Wong R., Todd H., Lasley B.L. (2004). Metabolic and endocrine effects of a polyunsaturated fatty acid-rich diet in polycystic ovary syndrome. J. Clin. Endocrinol. Metab..

[70] Barclay A.W., Petocz P., McMillan-Price J., Flood V.M., Prvan T., Mitchell P., Brand-Miller J.C. (2008). Glycemic index, glycemic load, and chronic disease risk—A meta-analysis of observational studies. Am. J. Clin. Nutr..

[71] Thorne M.J., Thompson L., Jenkins D. (1983). Factors affecting starch digestibility and the glycemic response with special reference to legumes. Am. J. Clin. Nutr..

[72] Shishehgar F., Tehrani F.R., Mirmiran P., Hajian S., Baghestani A.R., Moslehi N. (2016). Comparison of dietary intake between polycystic ovary syndrome women and controls. Glob. J. Health Sci..

[73] Douglas C.C., Norris L.E., Oster R.A., Darnell B.E., Azziz R., Gower B.A. (2006). Difference in dietary intake between women with polycystic ovary syndrome and healthy controls. Fertil. Steril..

[74] Altieri P., Cavazza C., Pasqui F., Morselli A.M., Gambineri A., Pasquali R. (2013). Dietary habits and their relationship with hormones and metabolism in overweight and obese women with polycystic ovary syndrome. Clin. Endocrinol. (Oxf.).

[75] Toscani M.K., Mario F.M., Radavelli-Bagatini S., Spritzer P.M. (2011). Insulin resistance is not strictly associated with energy intake or dietary macronutrient composition in women with polycystic ovary syndrome. Nutr. Res..

[76] Frost G., Dornhorst A., Caballero B., Allen L., Prentice A. (2012). Glycemic Index. Encyclopedia of Human Nutrition.

[77] Barr S., Hart K., Reeves S., Sharp K., Jeanes Y.M. (2011). Habitual dietary intake, eating pattern and physical activity of women with polycystic ovary syndrome. Eur. J. Clin. Nutr..

[78] Moran L.J., Ranasinha S., Zoungas S., McNaughton S.A., Brown W.J., Teede H.J. (2013). The contribution of diet, physical activity and sedentary behavior to body mass index in women with and without polycystic ovary syndrome. Hum. Reprod..

[79] Jang Y., Lee J.H., Kim O.Y., Park H.Y., Lee S.Y. (2001). Consumption of whole grain and legume powder reduces insulin demand, lipid peroxidation, and plasma homocysteine concentrations in patients with coronary artery disease randomized controlled clinical trial. Arterioscler. Thromb. Vasc. Biol..

[80] Jacobs D., Meyer K.A., Kushi L.H., Folsom A.R. (1998). Whole-grain intake may reduce the risk of ischemic heart disease death in postmenopausal women: The Iowa Women’s Health Study. Am. J. Clin. Nutr..

[81] Swennen K., Courtin C.M., Delcour J.A. (2006). Non-digestible oligosaccharides with prebiotic properties. Crit. Rev. Food Sci. Nutr..

[82] Baudrand R., Campino C., Carvajal C., Olivieri O., Guidi G., Faccini G., Vöhringer P., Cerda J., Owen G., Kalergis A. (2014). High sodium intake is associated with increased glucocorticoid production, insulin resistance and metabolic syndrome. Clin. Endocrinol. (Oxf.).

[83] Vedovato M., Lepore G., Coracina A., Dodesini A., Jori E., Tiengo A., Del Prato S., Trevisan R. (2004). Effect of sodium intake on blood pressure and albuminuria in Type 2 diabetic patients: The role of insulin resistance. Diabetologia.

[84] Aburto N.J., Hanson S., Gutierrez H., Hooper L., Elliott P., Cappuccio F.P. (2013). Effect of increased potassium intake on cardiovascular risk factors and disease: Systematic review and meta-analyses. BMJ.

[85] Jee S.H., Miller E.R., Guallar E., Singh V.K., Appel L.J., Klag M.J. (2002). The effect of magnesium supplementation on blood pressure: A meta-analysis of randomized clinical trials. Am. J. Hypertens..

[86] Altorf-van der Kuil W., Engberink M.F., Brink E.J., van Baak M.A., Bakker S.J., Navis G., van’t Veer P., Geleijnse J.M. (2010). Dietary protein and blood pressure: A systematic review. PLoS ONE.

[87] Tielemans S.M., Altorf-van Der Kuil W., Engberink M.F., Brink E.J., Van Baak M.A., Bakker S.J., Geleijnse J.M. (2013). Intake of total protein, plant protein and animal protein in relation to blood pressure: A meta-analysis of observational and intervention studies. J. Hum. Hypertens..

[88] Forman J.P., Rimm E.B., Stampfer M.J., Curhan G.C. (2005). Folate intake and the risk of incident hypertension among US women. JAMA.

[89] Xun P., Liu K., Loria C.M., Bujnowski D., Shikany J.M., Schreiner P.J., Sidney S., He K. (2012). Folate intake and incidence of hypertension among American young adults: A 20-y follow-up study. Am. J. Clin. Nutr..

[90] McCann S.E., Freudenheim J.L., Marshall J.R., Brasure J.R., Swanson M.K., Graham S. (2000). Diet in the epidemiology of endometrial cancer in western New York (United States). Cancer Causes Control.

[91] Xu W.H., Shrubsole M.J., Xiang Y.B., Cai Q., Zhao G.M., Ruan Z.X., Cheng J.R., Zheng W., Shu X.O. (2007). Dietary folate intake, MTHFR genetic polymorphisms, and the risk of endometrial cancer among Chinese women. Cancer Epidemiol. Biomark. Prev..

[92] Charalampakis V., Tahrani A.A., Helmy A., Gupta J.K., Singhal R. (2016). Polycystic ovary syndrome and endometrial hyperplasia: An overview of the role of bariatric surgery in female fertility. Eur. J. Obstet. Gynecol. Reprod. Biol..

[93] Otten J.J., Hellwig J.P., Meyers L.D. (2006). DRI, Dietary Reference Intakes: The Essential Guide to Nutrient Requirements.

[94] Moran L.J., Noakes M., Clifton P.M., Tomlinson L., Norman R.J. (2003). Dietary composition in restoring reproductive and metabolic physiology in overweight women with polycystic ovary syndrome. J. Clin. Endocrinol. Metab..

[95] Legro R.S. (2017). Effects of obesity treatment on female reproduction: Results do not match expectations. Fertil. Steril..

[96] Marsh K.A., Steinbeck K.S., Atkinson F.S., Petocz P., Brand-Miller J.C. (2010). Effect of a low glycemic index compared with a conventional healthy diet on polycystic ovary syndrome. Am. J. Clin. Nutr..

[97] Barr S., Reeves S., Sharp K., Jeanes Y.M. (2013). An isocaloric low glycemic index diet improves insulin sensitivity in women with polycystic ovary syndrome. J. Acad. Nutr. Diet..

[98] Knowler W.C., Barrett-Connor E., Fowler S.E., Hamman R.F., Lachin J.M., Walker E.A., Nathan D.M. (2002). Reduction in the Incidence of Type 2 Diabetes with Lifestyle Intervention or Metformin. N. Engl. J. Med..

[99] Orchard T.J., Temprosa M., Goldberg R., Haffner S., Ratner R., Marcovina S., Fowler S. (2005). The effect of metformin and intensive lifestyle intervention on the metabolic syndrome: The Diabetes Prevention Program randomized trial. Ann. Int. Med..

[100] Hawley J.A. (2004). Exercise as a therapeutic intervention for the prevention and treatment of insulin resistance. Diabetes Metab. Res. Rev..

[101] Vigorito C., Giallauria F., Palomba S., Cascella T., Manguso F., Lucci R., De Lorenzo A., Tafuri D., Lombardi G., Colao A. (2007). Beneficial effects of a three-month structured exercise training program on cardiopulmonary functional capacity in young women with polycystic ovary syndrome. J. Clin. Endocrinol. Metab..

[102] Brown A.J., Setji T.L., Sanders L.L., Lowry K.P., Otvos J.D., Kraus W.E., Svetkey P.L. (2009). Effects of exercise on lipoprotein particles in women with polycystic ovary syndrome. Med. Sci. Sports Exerc..

[103] Greaves C.J., Sheppard K.E., Abraham C., Hardeman W., Roden M., Evans P.H., Schwarz P., The IMAGE Study Group (2011). Systematic review of reviews of intervention components associated with increased effectiveness in dietary and physical activity interventions. BMC Public Health.

[104] Douglas C.C., Gower B.A., Darnell B.E., Ovalle F., Oster R.A., Azziz R. (2006). Role of diet in the treatment of polycystic ovary syndrome. Fertil. Steril..

[105] Hoeger K.M., Kochman L., Wixom N., Craig K., Miller R.K., Guzick D.S. (2004). A randomized, 48-week, placebo-controlled trial of intensive lifestyle modification and/or metformin therapy in overweight women with polycystic ovary syndrome: A pilot study. Fertil. Steril..

[106] Wu T., Gao X., Chen M., Van Dam R.M. (2009). Long-term effectiveness of diet-plus-exercise interventions vs. diet-only interventions for weight loss: A meta-analysis. Obes. Rev..

[107] Teede H.J., Joham A.E., Paul E., Moran L.J., Loxton D., Jolley D., Lombard C. (2013). Longitudinal weight gain in women identified with polycystic ovary syndrome: Results of an observational study in young women. Obesity.

[108] Domecq J.P., Prutsky G., Mullan R.J., Hazem A., Sundaresh V., Elamin M.B., Phung O.J., Wang A., Hoeger K., Pasquali R. (2013). Lifestyle modification programs in polycystic ovary syndrome: Systematic review and meta-analysis. J. Clin. Endocrinol. Metab..

[109] Thompson F.E., Kirkpatrick S.I., Subar A.F., Reedy J., Schap T.E., Wilson M.M., Krebs-Smith S.M. (2015). The national cancer institute’s dietary assessment primer: A resource for diet research. J. Acad. Nutr. Diet..

[110] McCarney R., Warner J., Iliffe S., van Haselen R., Griffin M., Fisher P. (2007). The Hawthorne Effect: A randomised, controlled trial. BMC Med. Res. Methodol..

